# Breadth of antibody activity elicited by an influenza B hemagglutinin vaccine is influenced by pre-existing immune responses to influenza B viruses

**DOI:** 10.1128/jvi.00705-25

**Published:** 2025-07-15

**Authors:** Michael A. Carlock, Spencer R. Pierce, Ted M. Ross

**Affiliations:** 1 Center for Vaccines and Immunology, University of Georgia 822673, Athens, Georgia, USA; 2 Florida Research and Innovation Center, Cleveland Clinic 587918 , Port Saint Lucie, Florida, USA; 3 Department of Infectious Diseases, University of Georgia 551782, Athens, Georgia, USA; 4 Department of Infection Biology, Lerner Research Institute, Cleveland Clinic 2569, Cleveland, Ohio, USA; Emory University School of Medicine, Atlanta, Georgia, USA

**Keywords:** influenza B virus, influenza vaccines, immunological imprinting, memory B cells, broadly-reactive antigens

## Abstract

Most people are exposed to influenza A and influenza B viruses (IBV) at an early age through natural infection or vaccination. These previous exposures can shape future immune responses and the ability to either recall memory B cells or generate *de novo* immune responses. Therefore, it is important to consider pre-existing immunity when testing vaccine candidates. The B/Victoria (B/VIC) and the B/Yamagata (B/YAM) lineages circulated in the human population between 1987 and 2020 until the B/YAM lineage stopped circulating following the coronavirus disease 2019 pandemic. Most people born prior to 2020 have pre-existing immune responses to both lineages of IBV. Therefore, in this report, the impact of vaccine-induced antibodies was explored in mice with pre-existing immune responses to B/YAM or B/VIC viruses alone or in combination. The recalled B cell responses induced by modern wild-type hemagglutinin (HA) vaccines from each IBV lineage were compared to the recalled B cell responses elicited by a broadly reactive antigen, B-COBRA-2 (BC2) HA, which elicits cross-lineage neutralizing antibodies. This report highlights the lineage specificity of immunological imprinting with IBV and demonstrates the antigen-specific activation of antibody-secreting B cells by broadly reactive antigens.

**IMPORTANCE:**

This report demonstrates how varying immunological backgrounds to influenza B virus influence vaccine-induced responses in mice. It further highlights that a broadly reactive influenza B virus hemagglutinin (HA) antigen stimulates higher levels of IgM-secreting cells compared to wild-type HA antigens.

## INTRODUCTION

Influenza viruses evolve to evade host neutralizing immune responses. There are two types of influenza viruses that primarily infect humans, influenza A virus (IAV) and influenza B virus (IBV). Seasonal influenza vaccination is currently the best method of protection against infection from influenza viruses. The viral surface glycoprotein hemagglutinin (HA), which is primarily responsible for viral attachment through recognition of sialic acid receptors on host epithelial cells, is the immunodominant viral protein driving host immune responses (1). Antibodies, which are the secreted forms of B cell receptors, can bind specifically to HA epitopes to prevent viral attachment on host cells and neutralize infection (2–4). B cells that secrete IgG are primarily responsible for this antigen-specific neutralization, but IgA and IgM antibody-secreting cells (ASCs) are also able to neutralize viruses (2–6). IgA, found in mucosal secretions like saliva or tears, can be secreted efficiently across mucosal epithelia for intracellular virus neutralization (2). During B cell development, the immunoglobulin (Ig) μ heavy chain protein is initially synthesized to form B cells with membrane-bound IgM (2, 5, 6). Therefore, IgM-secreting cells are the first antibody responses to viral infections and are especially important for early neutralization (2, 5, 6).

Immature B cells expressing IgM migrate from the bone marrow to peripheral tissues, such as the spleen and lymph nodes, where further maturation occurs (2). Most of these cells are known as follicular B cells and produce membrane-bound IgD in addition to IgM. However, a subset of these B cells, known as marginal zone (MZ) B cells, do not associate with IgD, but instead produce higher levels of the coreceptor CD21/CR2 that bind complement fragments (2). MZ and follicular B cells in the periphery are able to recognize viral antigens that result in activation and differentiation of these B cells into plasma cells that secrete antibodies of the same specificity as the antigen receptor (2). MZ B cells are limited to secreting IgM, but follicular B cells are able to secrete IgM or undergo isotype switching to replace surface IgM expression with IgG, IgA, or IgE (2, 7–10).

Infection or vaccination elicits B cells with specificity to epitopes on HA antigens so that upon future exposure to HA, long-lived plasma cells and memory B cells can rapidly respond (11–15). While most ASCs elicited in response to HA are directed against epitopes that are hypervariable, vaccination also boosts pre-existing memory B cell repertoires against conserved epitopes (16, 17). For IBV HA, the major antigenic epitopes surrounding the receptor-binding site consist of four major regions: the 120-loop (and surrounding regions), the 150-loop, the 160-loop, and the 190-helix (and surrounding 240-loop) (18). These sites have undergone strong selective pressure that led to the antigenic drift of influenza B viruses. IBV was first identified in 1940, but between 1987 and 1988, it diverged into two antigenically distinct lineages known as the B/Victoria (B/VIC) lineage and the B/Yamagata (B/YAM) lineage (19, 20). Early reports identified the 150-loop as an antigenic driver unique to B/YAM viruses (21), whereas the 160-loop was associated specifically for B/VIC viruses (22). However, both loops are now recognized as major antigenic sites on the IBV HA. The B/Victoria/2/1987 strain was the predominant cause of IBV infections in 1987 and was included in the seasonal influenza vaccine for the 1988–1989 influenza season (23). The following year, it was replaced by the B/Yamagata/16/1988 strain, and B/YAM-like viruses remained in the seasonal influenza vaccine until the 2001–2002 season (24). Beginning with the 2002–2003 season, one IBV strain was selected as the primary IBV representative to be included in the annual trivalent influenza virus vaccine with B/VIC-like strains selected eight times and B/YAM-like strains selected seven times (24). Beginning in 2012, the seasonal influenza virus vaccine was formulated as a quadrivalent vaccine with two influenza A strains, H1N1 and H3N2 subtypes, and two influenza B strains representing each lineage (25). In March 2020, at the start of the coronavirus disease 2019 (COVID-19) pandemic, there was no detection of B/YAM-like influenza B viruses circulating in the human population (26–28). This prompted the World Health Organization (WHO)’s influenza vaccine composition advisory committee in September 2023 to recommend a trivalent influenza vaccine that only included the B/VIC-like influenza B virus (29).

Most people are exposed to influenza viruses before the age of 7 through natural infection or vaccination (30). Therefore, it is important to understand how immune history might influence future immune responses to subsequent influenza virus exposures. The use of animal models with pre-existing immunity to influenza A viruses has been employed to assess influenza vaccines (31–40). However, few studies have utilized a pre-immune IBV animal model for the assessment of influenza B vaccines (41). Almost all people born prior to the start of the COVID-19 pandemic have pre-existing immune responses to both B/YAM and B/VIC influenza B viruses, so it is critical to understand how both B/VIC and B/YAM pre-immunity potentially influence future immune responses to IBV variants. In this report, the impact of vaccine-induced antibodies was assessed in sera collected from mice with pre-existing immune responses to historical B/YAM or B/VIC viruses alone or in combination. The recalled B cell responses induced by modern wild-type HA vaccines from each IBV lineage were compared to the immune responses elicited by a computationally optimized broadly reactive antigen (COBRA), B-COBRA-2 (BC2) HA, which elicits cross-lineage neutralizing antibodies (41). Overall, this study examined antibody and B cell immune profiles in mice with diverse pre-immune IBV backgrounds following vaccination with lineage-specific HA proteins versus an HA antigen that elicits cross-lineage neutralizing antibodies.

## MATERIALS AND METHODS

### Viruses and HA antigens

Influenza viruses were obtained through the Influenza Reagents Resource, BEI Resources, and the Centers for Disease Control and Prevention (CDC) or were provided by Sanofi Pasteur and Virapur, LLC (San Diego, CA, USA). Viruses were passaged once in 10-day-old embryonated, specific-pathogen-free chicken eggs per the protocol provided by the World Health Organization (42). IBVs of the Victoria lineage included the following strains: B/Hong Kong/330/2001 (B/HK/01), B/Brisbane/60/2008 (B/BR/08), B/Colorado/06/2017 (B/CO/17), and B/Washington/2/2019 (B/WA/19). IBVs of the Yamagata lineage included the following strains: B/Shanghai/361/2002 (B/SH/02), B/Massachusetts/02/2012 (B/MA/12), and B/Phuket/3073/2013 (B/PH/13).

### Vaccine preparation

The production of soluble, recombinant HA (rHA) proteins has been previously described (43). Briefly, HA sequences were truncated by removing the transmembrane domain and replacing it with a T4 Foldon trimerization domain, as well as an AviTag and a 6×His tag. These truncated sequences were cloned into a pcDNA3.1^+^ plasmid vector and transfected into human endothelial kidney 293T suspension cells for protein expression. Protein was purified by immobilized metal affinity chromatography, and protein concentration was assessed via bicinchoninic acid assay. Soluble, recombinant proteins were generated for B/PH/13-YAM HA, B/CO/17-VIC HA, and BC2 HA, as previously described (41).

### Viral infection and vaccination of mice

BALB/c mice (females, 6–8 weeks old) were purchased from The Jackson Laboratory (Bar Harbor, ME, USA). Mice were housed in microisolator units and allowed free access to food and water.

**Fig 1:**
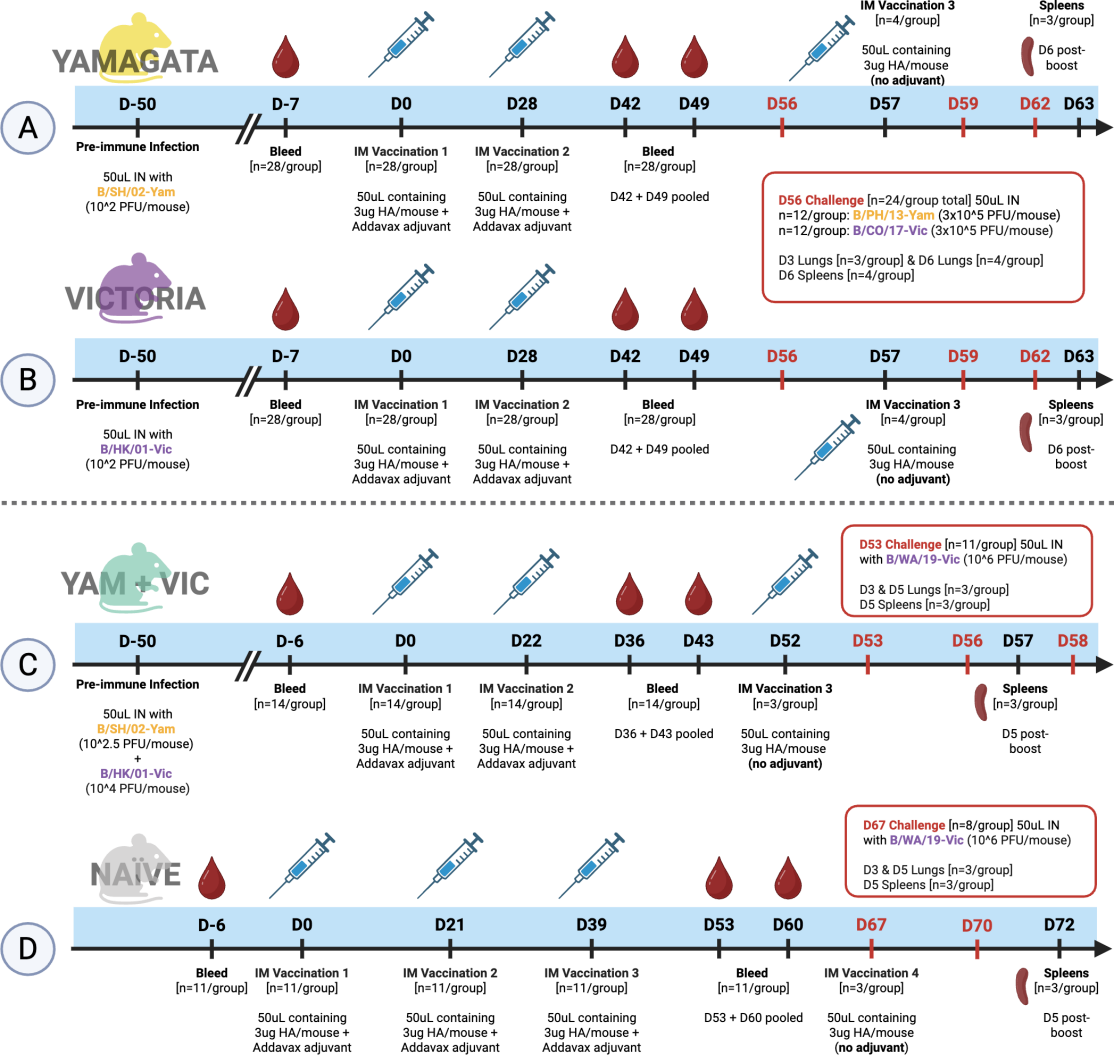
Naïve and IBV pre-immune mouse timeline. Mice were intranasally infected with 50 µL of a historical Yamagata lineage virus B/SH/02 (**A**) or Victoria lineage virus B/HK/01 (**B**) or 50 µL of a mixture of the two viruses (**C**). Mice were bled 43–44 days later to confirm pre-immune status via HAI and then intramuscularly vaccinated twice with 3 µg HA protein plus AddaVax adjuvant. Naïve mice (**D**) were instead vaccinated a third time. Mice were bled at 14 and 21 days post-boost, and serum was pooled between the time points to test HAI titers against a panel of viruses. A subset of mice was boosted again without adjuvant, and spleens were collected to measure ASCs via FluoroSpot. Mice that were initially pre-immunized with a single strain were divided in half and challenged with a modern strain representing the Victoria lineage (B/CO/17) or Yamagata lineage (B/PH/13). Naïve mice, and mice that were pre-immunized with both lineages, were challenged with a different drifted Victoria lineage virus (B/WA/19). Created in BioRender. Carlock, M. (2025) https://BioRender.com/v2mpn3m

Immunologically naïve mice were first pre-immunized to influenza B virus. Mice were anesthetized using 2%–3% vaporized isoflurane (Piramal Critical Care, Inc., Bethlehem, PA) and then intranasally infected with 50 µL of influenza B virus diluted in sterile phosphate buffered saline (PBS) using a historical strain representing the Victoria lineage (B/HK/01 at 10^2^ PFU/50 µL) (Fig. 1A), a historical strain representing the Yamagata lineage (B/SH/02 at 10^2^ PFU/50 µL) (Fig. 1B), or a mixture of the two (B/SH/02 at 10^2.5^ PFU/50 µL + B/HK/01 at 10^4^ PFU/50 µL) (Fig. 1C). Four weeks later, blood was collected from the facial vein, and serum was isolated. Seroconversion to these strains was confirmed for each mouse via hemagglutination inhibition (HAI) assay.

Mice were then intramuscularly immunized with 50 µL vaccines formulated with 3 µg of rHA mixed with AddaVax (InvivoGen, San Diego, CA, USA), an emulsified squalene-based oil-in-water emulsion adjuvant. Mock-vaccinated mice were administered a 50 µL dose divided in half with sterile PBS and AddaVax. Mice were boosted 3–4 weeks later with the same vaccines and then subsequently bled. To have a sufficient volume of serum to perform assays for individual mice instead of only pooled groups, mice were bled twice (at 14 and 21 days post-vaccination), and the isolated serum was instead pooled for the same mouse between these two timepoints. A week later, mice were divided into subsets and either challenged or immunized again with unadjuvanted vaccines containing the same antigens. Mice that were boosted again without adjuvant (*n* = 3/group) were sacrificed for spleens at day 5 or 6 post-vaccination. Spleens were collected in B cell media (1 L RPMI medium supplemented with 100 mL fetal bovine serum [FBS], 20 mL HEPES, 20 mL of a 50× minimum essential medium [MEM] amino acid solution, 10 mL of a 100× non-essential amino acid [NEAA] solution, 10 mL penicillin-streptomycin, 10 mL sodium pyruvate, 2 g of sodium bicarbonate, and 3.5 µL of 2-mercaptoethanol). Splenocytes were processed and stored in liquid nitrogen in freezing media (FBS with 1% dimethyl sulfoxide) until use.

Mice that were initially pre-immunized with a single strain were challenged with one of two influenza B viruses: a modern strain representing the Victoria lineage (B/CO/17 at 3 × 10^5^ PFU/50 µL) or a modern strain representing the Yamagata lineage (B/PH/13 at 3 × 10^5^ PFU/50 µL) (Fig. 1A and B). Lungs (*n* = 3–4/group) were collected 3 days post-infection (dpi) and 6 dpi to assess viral lung titers. Lungs were collected on dry ice and then stored at −80°C until use. Spleens (*n* = 4/group) were additionally taken at 6 dpi, collected in B cell media, processed, and stored in liquid nitrogen. Mice that were initially pre-immunized with a combination of the two strains (Fig. 1C) and naïve mice (Fig. 1D) were challenged with a newer strain representing the Victoria lineage (B/WA/19 at 1 × 10^6^ PFU/50 µL). Lungs (*n* = 3/group) were collected at 3 dpi and 5 dpi, and spleens (*n* = 3/group) were additionally collected at 5 dpi. Following any infection, mice were additionally assessed for weight loss, clinical signs, and mortality for up to 14 days.

### HAI assay

The HAI assay was used to assess functional antibodies to the HA that prevent the agglutination of avian red blood cells (RBCs) by IBVs. The protocols used for this assay were adapted from the WHO laboratory influenza virological surveillance manual (42). Sera samples were treated with receptor-destroying enzyme (RDE) (Denka, Seiken, Co., Japan) to inactivate nonspecific inhibitors, according to the manufacturer’s instructions prior to their use in the assay. In brief, three volumes of RDE were added to one volume of sera and incubated overnight at 37°C. The next day, the RDE was inactivated by incubating the samples at 56°C in a water bath for 30–60 min, after which six volumes of PBS were added to each sample, resulting in a final serum dilution of 1:10. RDE-treated sera were then diluted in a series of twofold serial dilutions in 96-well V-bottom plates, and an equal volume of influenza virus, adjusted to 8 hemagglutination units (HAU)/50 µL diluted in PBS, was added to each well of the plate. The plates were allowed to incubate at room temperature (RT) for 20 min. After incubation, 50 µL of a solution consisting of 0.8% turkey RBCs (Lampire Biologicals, Pipersville, PA, USA) diluted in PBS was added to each well. The plates were then mixed by gentle agitation and allowed to incubate for another 30 min at RT. After incubation with RBCs, the plates were tilted to observe the hemagglutination inhibition. The HAI antibody titer was determined by taking the reciprocal dilution of the last well that contained non-agglutinated RBCs. Positive control ferret reference serum was also included to confirm assay consistency between runs. Prior to use, the turkey RBCs were washed twice with 1× PBS, stored at 4°C, and used within 24 h of preparation. All viruses used in HAI assays were first treated with diethyl ether, as previously reported (44). Viruses were adjusted beforehand via HA assay to a concentration of 8 HAU/50 µL. The plates were covered and incubated at RT for 20 min, and then 0.8% turkey erythrocytes (Lampire Biologicals, Pipersville, PA, USA) in PBS were added. RBCs were prepared fresh each week, stored at 4°C, and used within 72 h of preparation. The plates were mixed by agitation and covered, and the RBCs settled for 30 min at RT. The HAI titer was determined by the reciprocal dilution of the last well that contained non-agglutinated RBCs. Positive and negative serum controls were included for each plate. Seroprotection was defined as an HAI titer ≥1:40, seronegativity as a titer less than 1:40, and seroconversion as a fourfold increase in titer compared to baseline, resulting in a titer of ≥1:40 (45).

### Anti-HA enzyme-linked immunosorbent assay (ELISA)

ELISAs were used to independently assess the elicited serum total IgG, total IgM, and total IgA antibody titers against a panel of influenza B HA antigens (B/HK/01, B/SH/02, B/PH/13, B/CO/17). In brief, each well of an Immulon 4HBX 96-well flat-bottom plate (Thermo Fisher, Waltham, MA, USA) was coated with 100 µL of carbonate buffer (pH 9.4) containing 5 mg/mL of bovine serum albumin (BSA) (Thermo Fisher, Waltham, MA, USA), and 500 ng/mL of rHA protein, as previously described (43). Plates were then incubated overnight at 4°C in a humidified chamber. The next day, the plates were decanted and blocked with 200 µL/well blocking buffer (1× PBS containing 5% BSA, 2% bovine gelatin, and 0.05% Tween-20) at 37°C for 90 min in a humidified chamber. Serum samples were serially diluted twofold in blocking buffer starting at a dilution of 1:500 for IgG or 1:50 for IgM and IgA. Plates were then incubated overnight at 4°C in a humidified chamber. The next day, the plates were washed five times with wash buffer (1× PBS, 0.05% Tween-20). The wash buffer was then decanted, and secondary antibody diluted 1:4,000 in blocking buffer was added to each well. The following horseradish peroxidase (HRP)-conjugated antibodies from Southern Biotech (Birmingham, AL, USA) were used: Goat Anti-Mouse IgG (cat #1030-05), Goat Anti-Mouse IgG1 (cat #1070-05), and Goat Anti-Mouse IgM (cat #1021-05). The plates were incubated at 37°C for 90 min in a humidified chamber and then washed five times with wash buffer. The plates were then decanted, and 100 µL/well of ABTS Diammonium Salt (Amersco, Solon, Ohio, USA) working solution was added to each plate. Plates were incubated at 37°C for 15 min, and then 50 µL/well of a 1% sodium dodecyl sulfate solution was added to each well to stop the colorimetric reaction. Optical density values of each well were measured at 414 nm using a spectrophotometer (PowerWave XS, Agilent Technologies, Santa Clara, CA, USA) and plotted in GraphPad Prism (San Diego, CA, USA) for each dilution point. Total area-under-the-curve (AUC) and standard error were then calculated using GraphPad Prism.

### FluoroSpot assay

The FluoroSpot assay is a fluorescent alternative to the Enzyme Linked Immunosorbent Spot used to measure B cell responses from splenocytes collected post-vaccination. The protocols used for this assay were adapted from Cellular Technology Limited (CTL; Cleveland, OH, USA) with helpful tips from Mabtech (46). Mouse IgA/IgG/IgM Three-Color FluoroSpot kits from CTL were used. First, previously processed splenocytes were removed from LN_2_ on dry ice, quickly thawed in a 37°C water bath, and transferred to 15 mL conical tubes filled with 10 mL of pre-warmed B cell media. Cells were washed three times through centrifugation at 300 × *g* for 10 min followed by gentle cell pellet resuspension in B cell media. After the third wash, samples were placed in a 37°C + 5% CO_2_ incubator for 1 h. Cells were resuspended after resting in the incubator, and any aggregated cell debris was allowed to sediment for ~1 min. The cell suspension without the debris was carefully transferred to 14 mL polystyrene round-bottom tubes, and the volume was adjusted to 10 mL with B cell media. Live cell counts were recorded using an automated cell counter with trypan blue, and cell counts were confirmed to be between 1 and 4 million cells/mL. A total of 10 µL of Mouse B-Poly-S polyclonal B cell stimulator (included in CTL FluoroSpot kit) was added to each sample. Cells were then incubated in a 37°C + 5% CO_2_ incubator for 4 days. On the 3rd day, plates were coated. Briefly, clear 96-well plates with polyvinylidene difluoride (PVDF) membranes (included in CTL FluoroSpot kit) were treated by adding 20 µL/well of 70% ethanol and quickly rinsing five times with sterile water. Total Ig plates were coated with 80 µL/well of capture solution containing anti-mouse Igκ and anti-mouse Igλ, per the included CTL kit protocol. Separate antigen-specific plates were instead coated with 50 µL/well of beta-propiolactone (BPL)-inactivated virus (B/HK/01, B/SH/02, B/PH/13, B/CO/17), titrated to 64 HAU. Plates were wrapped in foil and incubated overnight at 4°C. The following day, plates were washed three times with sterile PBS and then loaded with 200 µL/well of B cell media to incubate at RT while the cells are prepared. Cells were removed from the incubator, resuspended, and transferred to new 15 mL conical tubes. Samples were washed twice through centrifugation at 300 × *g* for 10 min followed by gentle cell pellet resuspension in B cell media. Live cell counts were recorded using an automated cell counter with trypan blue, and volumes were adjusted for each sample to bring cell counts to 3 × 10^6^ cells/mL. Cells were added to deep-well blocks in triplicate for each sample, and twofold serial dilutions were performed. The B cell media in the FluoroSpot plates was dumped, and 100 µL/well of fresh B cell media was added. A total of 100 µL of each dilution block (300,000 initial cell dilution) was transferred to antigen-specific plates. For total Ig plates, the dilution blocks were diluted 1:5 with B cell media (60,000 initial cell dilution). Plates were wrapped in aluminum foil and placed in a 37°C + 5% CO_2_ incubator for 16–18 h. Afterward, plates were washed two times with sterile PBS containing 0.1% Triton X-100 and then washed four times with sterile PBS only. A total of 60 µL/well of Detection Solution (anti-mouse IgG conjugated with Hapten_1_, anti-mouse IgM conjugated with fluorescein isothiocyanate (FITC), and anti-mouse IgA conjugated with biotin), filtered through a 0.22 µm filter unit, was added to each plate. Plates were wrapped in foil to incubate for 2 h at RT in the dark. Plates were then washed two times with sterile PBS containing 0.1% Triton X-100 and then two times with sterile PBS. A total of 60 µL/well of Tertiary Solution (anti-Hapten CTL-Yellow for IgG, anti-FITC Alexa Fluor 488 for IgM, and SA-CTL-Red for IgA), filtered through a 0.22 µm filter unit, was added to each plate. Plates were wrapped in foil to incubate for 1 h at RT in the dark and then washed two times with sterile water. The plate underdrains were removed, and the plates were washed once more with sterile water. Plates were allowed to dry at RT in the dark for 2 days and then scanned and counted using a CTL ImmunoSpot Analyzer with ImmunoSpot Fluoro-X Software which creates a data output of spot counts and additional spot info. For each individual replicate, the number of spots in a well was multiplied by the dilution factor (1 million cells divided by the number of input cells; i.e., 1 M / 300,000 cells = 3.33×) to yield spot-forming units (SFUs) per 1 million input cells. For this analysis, the four dilutions were averaged together for each individual replicate.

### Viral plaque assay

MDCK cells were seeded at a concentration of 1 × 10^6^ cells/well into six-well plates on day 1. The following day, after MDCK cells reached ~90% confluency in each well, the plates were washed three times with Dulbecco’s modified Eagle medium supplemented with 1% penicillin-streptomycin (DMEM + P/S). Lung samples were thawed on ice, homogenized, and then serially diluted 10-fold in DMEM + P/S. Media was removed from the plates, and 100 µL of the viral dilutions was added. The plates were incubated at RT for 1 h and gently rocked every 15 min. Following the incubation, the plates were washed twice with DMEM + P/S. Following the second wash, an overlay solution of plaque media (2× MEM, HEPES, L-glutamine, P/S, sodium bicarbonate, and 1 µg/mL of N-tosyl-L-phenylalanine chloromethyl ketone [TPCK]-treated trypsin) mixed 1:1 with 1.6% agarose was added to each well. Plates were then incubated at 37°C + 5% CO_2_ for 72 h. Afterward, the gel overlays were removed, the cells were fixed with 10% buffered formalin for 10 min, and then cells were stained with 1% crystal violet for 10 min. Plates were then rinsed thoroughly with fresh water to remove excess crystal violet, and plates were allowed to air dry for at least 24 h. Plaques were then counted, and the viral titer (PFU per milliliter) for each sample was calculated using the number of plaque colonies and the dilution factor.

### Statistical analysis

Statistical significance was defined as a *P*-value < 0.05. GraphPad Prism version 10 software was used throughout. For HAI, paired parametric *t*-tests with Holm-Šídák multiple comparisons test were performed to determine the significance of vaccine-induced antibodies between timepoints against each strain for each pre-immune background. Ordinary one-way analysis of variance (ANOVA) with Tukey’s multiple comparison test was performed to determine the significance of HAI fold changes between each vaccination group and for FluoroSpot analysis, comparing the mean of each column. For weight loss curves, a mixed-effects model with the Geisser-Greenhouse correction and Tukey’s multiple comparison test was utilized. In all cases, ns = *P* > 0.05, * = *P* ≤ 0.05, ** = *P* ≤ 0.01, *** = *P* ≤ 0.001, and **** = *P* ≤ 0.0001.

## RESULTS

### Vaccine-induced serological responses in naïve and pre-immune mice

**Fig 2:**
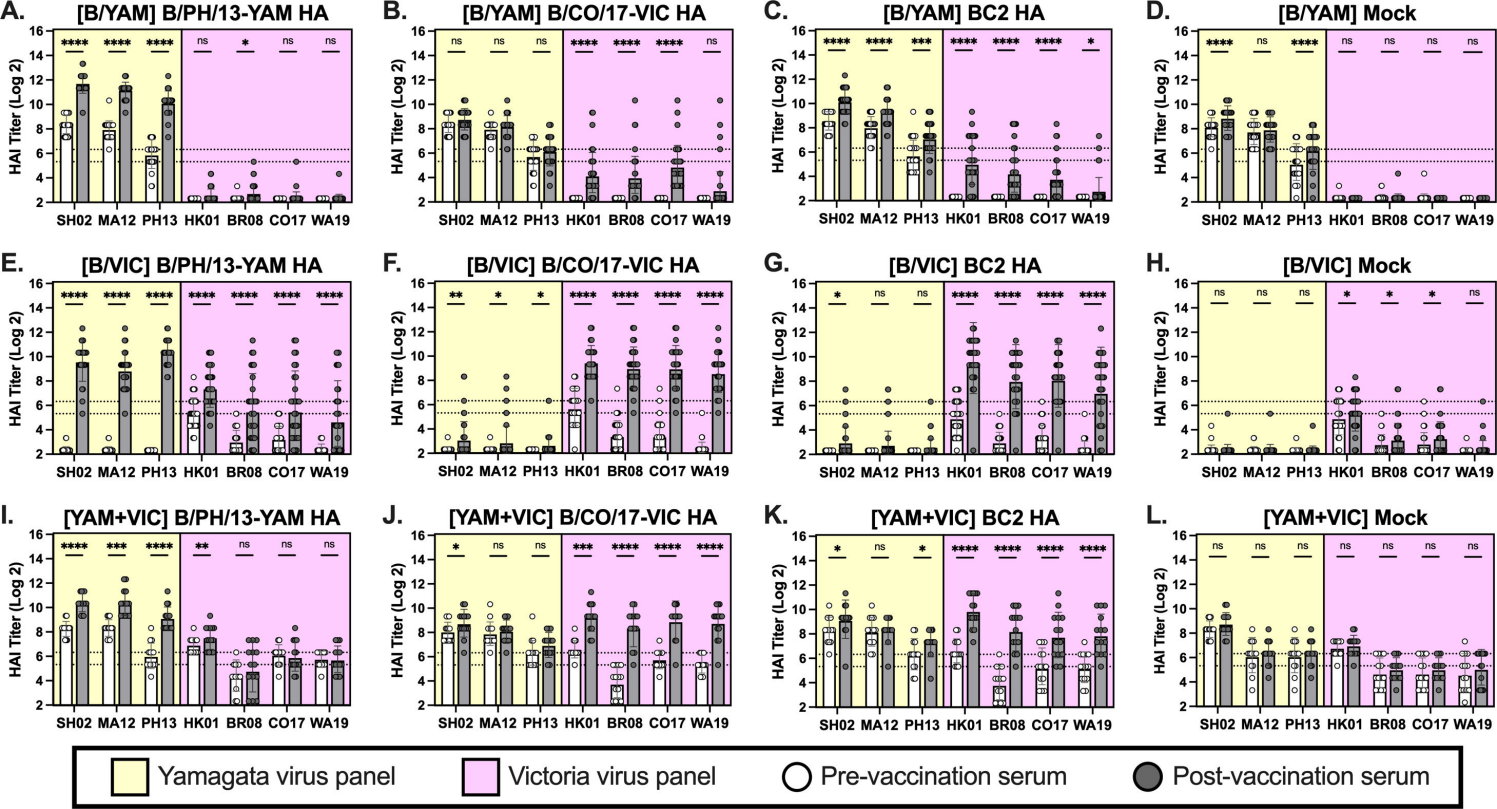
Pre- and post-vax HAI results. Serum collected before vaccination (white bars) and after vaccination (gray bars) was assayed against a panel of Yamagata lineage viruses (yellow background) and Victoria lineage viruses (pink background). Mice pre-immunized with SH/02-YAM (**A–D**), or HK/01-VIC (**E–H**), or SH/02-YAM + HK/01-VIC (**I–L**) were vaccinated with the B/YAM WT strain B/PH/13 (**A, E, I**), the B/VIC WT strain B/CO/17 (**B, F, J**), BC2 (**C, G, K**), or an adjuvanted PBS mock (**D, H, L**). Log2 HAI titers for individual mice are shown as scatter dot plots with bars representing the geometric mean titer of each group and error bars indicating the geometric standard deviation. Dotted lines indicate a 1:40 to 1:80 HAI titer range. Statistical analyses were performed using paired parametric *t*-tests to determine the significance of vaccine-induced antibodies between time points.

Following pre-immunization, mice rested for ~50 days prior to vaccination with either BC2 HA, B/PH/13-YAM HA, B/CO/17-VIC HA, or PBS (mock) mixed with AddaVax adjuvant. (Fig. 1). Mice pre-immunized to B/YAM (B/SH/02) had high baseline serum HAI titers against the panel of B/YAM influenza viruses (yellow), but there were no detectable HAI titers against the panel of B/VIC influenza viruses (violet) (Fig. 2A through D). Immunization with the B/PH/13-YAM HA boosted serum HAI titers against the lineage-matched influenza viruses, but not the B/VIC influenza viruses (Fig. 2A). Mice vaccinated with the B/CO/17-VIC HA had similar HAI titers post-vaccination against the panel of B/YAM influenza viruses as pre-vaccination HAI titers (Fig. 2B) but had HAI titers that significantly increased against most of the B/VIC influenza viruses post-vaccination, with an average Log2 fold change of 1.24–2.08 (Table 1). Mice vaccinated with BC2 HA had statistically significant fold rises in HAI titers against all viruses in the panel post-vaccination (Fig. 2C) with an average Log2 fold change of 1.16–1.25 against the B/YAM influenza viruses and 1.17–2.14 against the B/VIC influenza viruses (Table 1). B/YAM pre-immune mice vaccinated with B/CO/17-VIC HA and BC2 HA had significant increases in HAI titers against the panel of B/VIC influenza viruses; however, the majority of the mice had HAI titers below the seroprotective (HAI ≥ 1:40, or Log2 of 5.32) level (Table 2). Some B/YAM pre-immune mock-vaccinated mice had an increase in HAI titers against two of the B/YAM influenza viruses (Fig. 2D).

**TABLE 1:** Average Log2 HAI geometric mean titer and fold change^*a*^

Pre-immunity	Vaccination	B/Yamagata virus panel									B/Victoria virus panel											
		B/SH/02			B/MA/12			B/PH/13			B/HK/01			B/BR/08			B/CO/17			B/WA/19		
		Pre-vax	Post-vax	Fold	Pre-vax	Post-vax	Fold	Pre-vax	Post-vax	Fold	Pre-vax	Post-vax	Fold	Pre-vax	Post-vax	Fold	Pre-vax	Post-vax	Fold	Pre-vax	Post-vax	Fold
B/YAM	PH/13 (*n* = 29)	8.36	11.68	1.40	7.91	11.13	1.41	5.84	10.07	**1.73**	2.32	2.55	1.10	2.35	2.69	1.14	2.32	2.42	1.04	2.32	2.37	1.02
	CO/17 (*n* = 29)	8.39	8.73	1.04	7.92	8.14	1.03	5.69	6.10	1.07	2.32	4.09	**1.76**	2.32	3.94	**1.70**	2.32	4.82	**2.08**	2.32	2.88	1.24
	BC2 (*n* = 29)	8.53	10.11	1.18	7.96	9.27	1.16	5.64	7.04	1.25	2.32	4.96	**2.14**	2.32	4.16	**1.79**	2.32	3.72	**1.60**	2.32	2.72	1.17
	Mock (*n* = 29)	8.08	8.82	1.09	7.70	7.88	1.02	5.05	6.16	1.22	2.35	2.32	0.99	2.38	2.37	1.00	2.37	2.32	0.98	2.32	2.32	1.00
B/VIC	PH/13 (*n* = 29)	2.38	9.53	**4.01**	2.38	8.79	**3.69**	2.32	10.31	**4.44**	5.25	7.32	1.39	2.95	5.42	**1.84**	3.18	5.42	**1.71**	2.47	4.61	**1.87**
	CO/17 (*n* = 29)	2.35	3.05	1.30	2.35	2.83	1.20	2.32	2.61	1.13	5.63	9.40	**1.67**	3.33	8.94	**2.68**	3.30	8.92	**2.70**	2.45	8.51	**3.48**
	BC2 (*n* = 29)	2.32	2.91	1.25	2.32	2.71	1.17	2.32	2.54	1.09	4.87	9.46	**1.94**	2.90	7.94	**2.74**	3.24	8.03	**2.48**	2.54	6.97	**2.74**
	Mock (*n* = 28)	2.40	2.39	0.99	2.38	2.39	1.00	2.35	2.37	1.01	4.86	5.32	1.09	2.75	3.12	1.13	2.79	3.23	1.16	2.35	2.49	1.06
YAM + VIC	PH/13 (*n* = 14)	8.23	10.37	1.26	8.30	10.34	1.25	5.95	9.06	**1.52**	6.87	7.48	1.09	4.23	4.73	1.12	6.06	5.85	0.97	5.71	5.65	0.99
	CO/17 (*n* = 14)	8.00	8.68	1.09	7.84	8.06	1.03	6.24	6.89	1.10	6.57	9.18	1.40	3.70	8.32	**2.25**	5.70	8.84	**1.55**	5.35	8.70	**1.63**
	BC2 (*n* = 14)	8.26	9.07	1.10	8.10	8.25	1.02	6.20	7.24	1.17	6.54	9.80	**1.50**	3.74	8.16	**2.18**	5.12	7.69	**1.50**	5.11	7.81	**1.53**
	Mock (*n* = 14)	8.30	8.70	1.05	6.03	6.32	1.05	6.03	6.32	1.05	6.72	6.92	1.03	4.60	4.96	1.08	4.60	4.96	1.08	4.52	4.99	1.11
Naïve	PH/13 (*n* = 11)	2.32	6.76	**2.91**	2.32	7.80	**3.36**	2.32	9.98	**4.30**	2.32	2.32	1.00	2.32	2.32	1.00	2.32	2.32	1.00	2.32	2.32	1.00
	CO/17 (*n* = 11)	2.32	2.32	1.00	2.32	2.32	1.00	2.32	2.32	1.00	2.32	3.88	1.67	2.32	3.51	1.51	2.32	3.93	1.69	2.32	3.75	1.62
	BC2 (*n* = 11)	2.32	2.54	1.10	2.32	2.58	1.11	2.32	2.54	1.10	2.32	3.92	1.69	2.32	3.50	1.51	2.32	2.65	1.14	2.32	2.74	1.18
	Mock (*n* = 11)	2.32	2.32	1.00	2.32	2.32	1.00	2.32	2.32	1.00	2.32	2.32	1.00	2.32	2.32	1.00	2.32	2.32	1.00	2.32	2.32	1.00

^*a*^ Fold values ≥1.50 are shown in bold.

**TABLE 2:** Percent seroprotection at each time point and seroconversion between time points*^a^*^,^*^b^*^,*c*^

Pre-immunity	Vaccination	B/Yamagata virus panel									B/Victoria virus panel											
		B/SH/02			B/MA/12			B/PH/13			B/HK/01			B/BR/08			B/CO/17			B/WA/19		
		Pre-SP (%)	Post-SP (%)	SC (%)	Pre-SP (%)	Post-SP (%)	SC (%)	Pre-SP (%)	Post-SP (%)	SC (%)	Pre-SP (%)	Post-SP (%)	SC (%)	Pre-SP (%)	Post-SP (%)	SC (%)	Pre-SP (%)	Post-SP (%)	SC (%)	Pre-SP (%)	Post-SP (%)	SC (%)
B/YAM	PH/13 (*n* = 29)	100	100	100	100	100	100	90	100	**100**	0	0	0	0	3	3	0	3	3	0	0	0
	CO/17 (*n* = 29)	100	100	3	100	100	0	83	90	14	0	31	**31**	0	28	**28**	0	48	**48**	0	14	14
	BC2 (*n* = 29)	100	100	48	100	100	34	72	97	41	0	55	**55**	0	38	**38**	0	21	**21**	0	17	17
	Mock (*n* = 29)	100	100	21	100	100	0	59	90	24	0	0	0	0	0	0	0	0	0	0	0	0
B/VIC	PH/13 (*n* = 29)	0	100	**100**	0	100	**100**	0	100	**100**	62	97	59	7	62	**59**	10	59	**55**	0	48	**48**
	CO/17 (*n* = 29)	0	17	17	0	17	17	0	7	7	76	100	**90**	14	100	**100**	10	100	**100**	3	100	**100**
	BC2 (*n* = 29)	0	17	17	0	14	14	0	3	3	59	97	**97**	3	97	**97**	14	97	**97**	3	79	**79**
	Mock (*n* = 28)	0	4	0	0	4	4	0	0	0	50	75	4	7	14	11	7	11	4	0	4	4
YAM + VIC	PH/13 (*n* = 14)	100	100	79	100	100	64	93	100	**93**	100	100	7	36	57	36	93	86	7	93	79	7
	CO/17 (*n* = 14)	100	100	21	100	100	14	100	100	21	100	100	79	29	100	**93**	93	100	**93**	79	100	**86**
	BC2 (*n* = 14)	100	100	29	100	100	7	86	93	36	100	100	**93**	29	100	**100**	64	100	**86**	64	100	**93**
	Mock (*n* = 14)	100	100	0	86	93	0	86	93	0	100	100	0	43	57	0	43	57	0	50	64	14
Naïve	PH/13 (*n* = 11)	0	82	**82**	0	100	**100**	0	100	**100**	0	0	0	0	0	0	0	0	0	0	0	0
	CO/17 (*n* = 11)	0	0	0	0	0	0	0	0	0	0	45	45	0	36	36	0	45	45	0	36	36
	BC2 (*n* = 11)	0	9	9	0	9	9	0	9	9	0	45	45	0	27	27	0	9	9	0	9	9
	Mock (*n* = 11)	0	0	0	0	0	0	0	0	0	0	0	0	0	0	0	0	0	0	0	0	0

^*a*^ Pre-SP, seroprotection (HAI ≥ 1:40) at pre-vaccination time point.

^*b*^ Post-SP, seroprotection (HAI ≥ 1:40) at post-vaccination time point.

^*c*^ SC, seroconversion (HAI ≥ 1:40 at post-vaccination time point and ≥4-fold increase); groups with ≥50 % SC are shown in bold.

Mice pre-immunized to B/VIC (B/HK/01) influenza virus had little-to-no serum HAI antibody titers against the panel of B/YAM influenza viruses, but higher pre-vaccination HAI titers against the panel of B/VIC influenza viruses (Fig. 2E–H). Despite low baseline HAI titers against the panel of B/YAM influenza viruses, all mice vaccinated with B/PH/13-YAM HA were boosted to seroprotective levels (Fig. 2E) with an average Log2 fold change of 3.69–4.44 (Table 1). Interestingly, mice vaccinated with B/PH/13-YAM HA also had significantly higher HAI titers against the panel of B/VIC influenza viruses, with an average Log2 fold change of 1.39–1.87 (Table 1). Additionally, most mice (97%) had a seroprotective HAI titer against the pre-immunization virus B/HK/01, and many mice (48%–62%) had seroprotective HAI titers against the other B/VIC viruses (Table 2). All mice vaccinated with B/CO/17-VIC HA elicited seroprotective HAI titers against the panel of B/VIC influenza viruses (Fig. 2F), but only a few mice (7%–17%) had seroprotective HAI titers against the B/YAM influenza viruses (Table 2). In general, mice vaccinated with BC2 HA had similar serum HAI titers as mice vaccinated with B/CO/17-VIC HA; however, six mice did not have seroprotective HAI titers against B/WA/19 (B/VIC) (Fig. 2G). Some B/VIC pre-immune mock-vaccinated mice had increased serum HAI titers against the B/VIC influenza viruses (Fig. 2H).

Mice pre-immunized with historical influenza B viruses from both lineages had high baseline titers against all viruses in both panels (Fig. 2I through L). All mice had seroprotective titers to both pre-immune viruses, B/SH/02 (B/YAM) and B/HK/01 (B/VIC), following infection; however, the average Log2 HAI titer to B/SH/02 (B/YAM) was 1.23 times higher than the average Log2 HAI titer to B/HK/01 (B/VIC) (Table 1). In general, most mice (86%–100%) had seroprotective baseline titers to the other B/YAM influenza viruses (Table 2) with an HAI Log2 geometric mean titer (GMT) of 5.95–8.30. In contrast, fewer mice (29%–93%) had seroprotective baseline titers to the other B/VIC influenza viruses (Table 2) with an HAI Log2 GMT of 3.70–6.06. Mice vaccinated with B/PH/13-YAM HA had significantly boosted HAI titers against the panel of B/YAM influenza viruses but no significant increases against the panel of B/VIC influenza viruses except the B/HK/01 pre-immunizing virus (Fig. 2I). Similarly, mice vaccinated with B/CO/17-VIC HA had significantly boosted HAI titers against the panel of B/VIC viruses, but no statistically significant increases against the B/YAM influenza viruses except the B/SH/02 (B/YAM) pre-immunizing virus (Fig. 2J). Mice vaccinated with BC2 HA had similar post-vaccination HAI titers as mice vaccinated with B/CO/17-VIC HA but had significantly increased HAI titers against B/PH/13 (B/YAM) virus and a greater increase to B/HK/01 (B/VIC) (Fig. 2K). Mock-vaccinated mice had no statistically significant increases in post-vaccination HAI titers (Fig. 2L). Compared to B/VIC pre-immune mice, pre-immunization with both B/SH/02 (B/YAM) and B/HK/01 (B/VIC) reduced the average fold changes in HAI titers induced by B/CO/17-VIC HA or BC2 HA vaccination against the B/CO/17 (B/VIC) and B/WA/19 (B/VIC) viruses by 40%–53%, and reduced the average fold changes in HAI titers against B/HK/01 (B/VIC) and B/BR/08 (B/VIC) by 16%–23% (Table 1). Regardless, 100% of these B/SH/02 (B/YAM) and B/HK/01 (B/VIC) pre-immune mice had seroprotective HAI titers following vaccination against all B/VIC viruses. Including the B/YAM virus in the pre-immunization regimen increased (~93%) the seroprotective levels against the B/YAM viruses compared to mice pre-immunized with B/VIC alone (Table 2).

Naïve mice vaccinated with B/PH/13-YAM HA elicited robust HAI titers to the B/YAM influenza virus panel (Table 1). In general, however, mice that were immunologically naive prior to vaccination had low HAI titers (Table 1). Mice vaccinated with B/CO/17-VIC HA had statistically significant increases in HAI titers against the panel of B/VIC influenza viruses (Table 1), but less than half of mice had seroprotective HAI titers (Table 2). Without pre-existing anti-influenza immune responses prior to vaccination, mice vaccinated with wild-type antigens had no increased serum HAI titers against the panel of viruses in the opposite lineage (Table 1). Naïve mice vaccinated with BC2 HA had a significant increase in HAI titers against B/HK/01 (B/VIC) influenza virus (Table 1), but, in general, had minor increases in HAI titers post-vaccination, except for one mouse with seroprotective titers against all influenza B viruses (Table 2). Naïve mock mice had no detectable HAI titers (Table 1).

Post-vaccination serum samples were also assessed for anti-HA-specific IgG, IgM, and IgA via ELISA (Fig. S1). All groups of mice had predominantly anti-HA IgG serum antibodieswith an area-under-the curve (AUC) measurement >6. Mice vaccinated with B/PH/13-YAM HA had an average AUC value of 12.37 across the different immunological backgrounds, ranging from ~10 for naïve mice to >14 for mice pre-immunized with B/SH/02 (B/YAM) virus (Fig. S1A). In contrast, mice vaccinated with B/PH/13-YAM HA had the lowest average AUC (8.56) among the vaccine groups, whereas mice vaccinated with BC2 HA had the highest average AUC (9.15) that ranged from ~6 for naïve mice to >11 for mice pre-immunized with B/HK/01 (B/VIC) virus (Fig. S1B). Overall, pre-immune mice had similar levels of anti-HA IgG-secretion against the two B/YAM rHA proteins, with high serum titers elicited following vaccination with B/PH/13-YAM HA or BC2 HA (Fig. S1A and C). Likewise, pre-immune mice had similar levels of anti-HA IgG secretion against the two B/VIC rHA proteins with high titers in mice following vaccination with B/CO/17-VIC HA or BC2 HA (Fig. S1B and D). Anti-HA IgM and IgA serum antibodies were less abundant compared to anti-HA IgG. Similar antibody patterns were observed for serum anti-HA IgM across all rHA proteins, with the highest values generally observed in naïve mice and mice vaccinated with B/PH/13-YAM HA or BC2 HA (Fig. S1E through H). Low levels of IgA were elicited in pre-immune mice, and little-to-no anti-HA IgA was detected in naïve mice (Fig. S1I through L).

Overall, pre-existing immunity greatly influences how effective influenza vaccines are in generating serum antibody responses. In general, mice pre-immune to influenza B virus have increased serum antibody titers compared to mice naïve to influenza (Tables 1 and 2). Imprinting to IBV can, however, positively or negatively influence subsequent influenza vaccinations in mice. Mice imprinted with the B/VIC lineage and vaccinated with B/PH/13-YAM HA generated robust HAI titers against the B/VIC virus panel without reducing the ability to mount substantial titers to the B/YAM virus panel (Fig. 2E). In contrast, B/YAM pre-immune mice immunized with B/CO/17-VIC HA did not have increased titers to the B/YAM virus panel beyond baseline serological levels and had reduced titers to the B/VIC virus panel (Fig. 2B) compared to mice with pre-immune backgrounds containing B/VIC (Fig. 2F and J). Vaccination with BC2 HA elicited similar serological responses as B/CO/17-VIC HA but also increased responses to the B/YAM virus panel in mice pre-immunized to B/YAM (Fig. 2C).

### Immunoglobulin profiles from antigen-secreting B cells following HA vaccination

**Fig 3:**
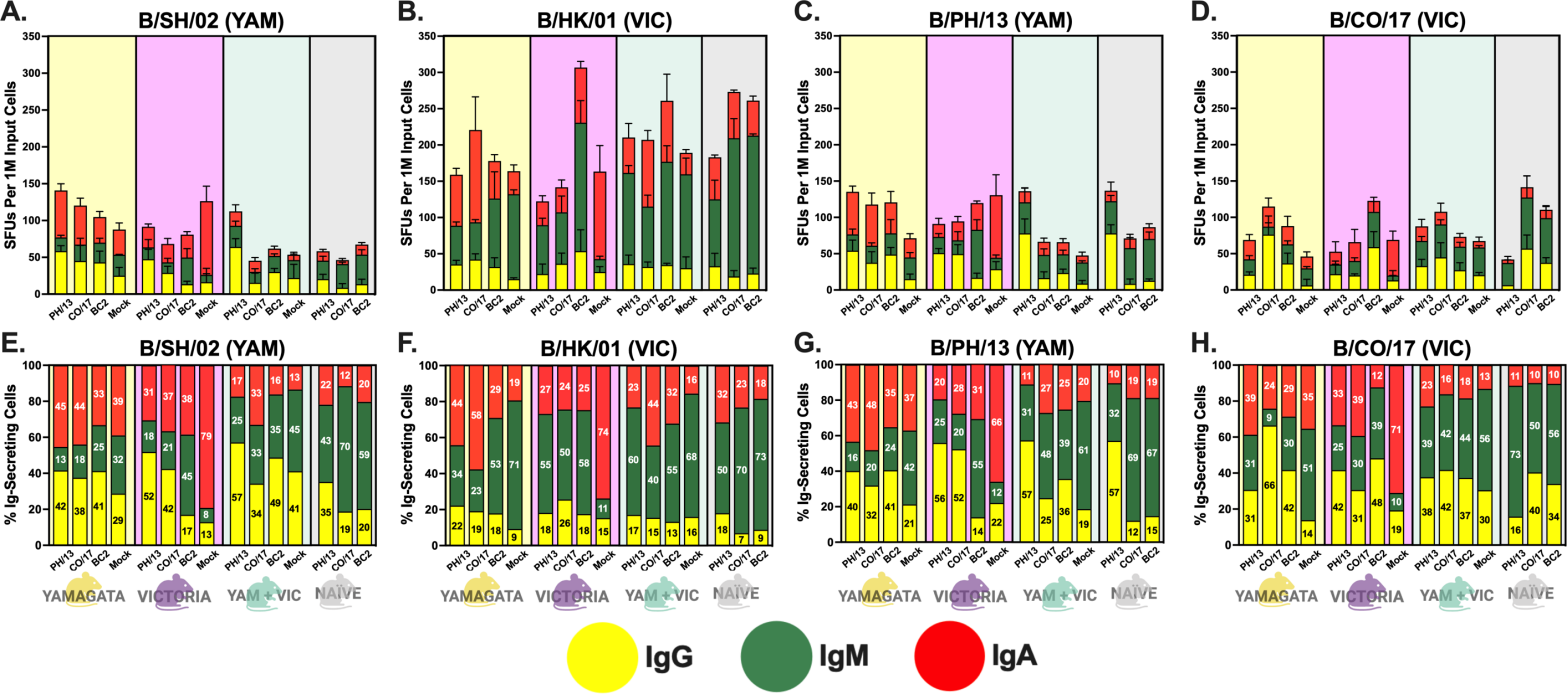
Antigen-specific IgG, IgM, and IgA. Three-Color FluoroSpots measuring IgG (yellow bars), IgM (green bars), and IgA (red bars) were run using splenocytes from mice pre-immunized with B/SH/02-YAM (yellow background), B/HK/01-VIC (violet background), or B/SH/02-YAM + B/HK/01-VIC (light blue background), or mice that were influenza naïve (gray background). The mean SFUs of three replicates are shown with an error bar for standard deviation (**A–D**), as well as the percentage of Ig-secreting cells (**E–H**) for IgG (yellow bars), IgM (green bars), and IgA (red bars). Mice are vaccinated with either B/PH/13, B/CO/17, BC2, or adjuvanted PBS mock. Plates were coated with BPL-inactivated viruses representing the pre-immune strains, B/SH/02-YAM (**A, E**) and B/HK/01-VIC (**B, F**), or the more modern WT comparators, B/PH/13-YAM (**C, G**) and B/CO/17-VIC (**D, H**). Statistical analyses were performed on the SFUs using ordinary one-way ANOVA with Tukey’s multiple comparison test (File S4).

**Fig 4:**
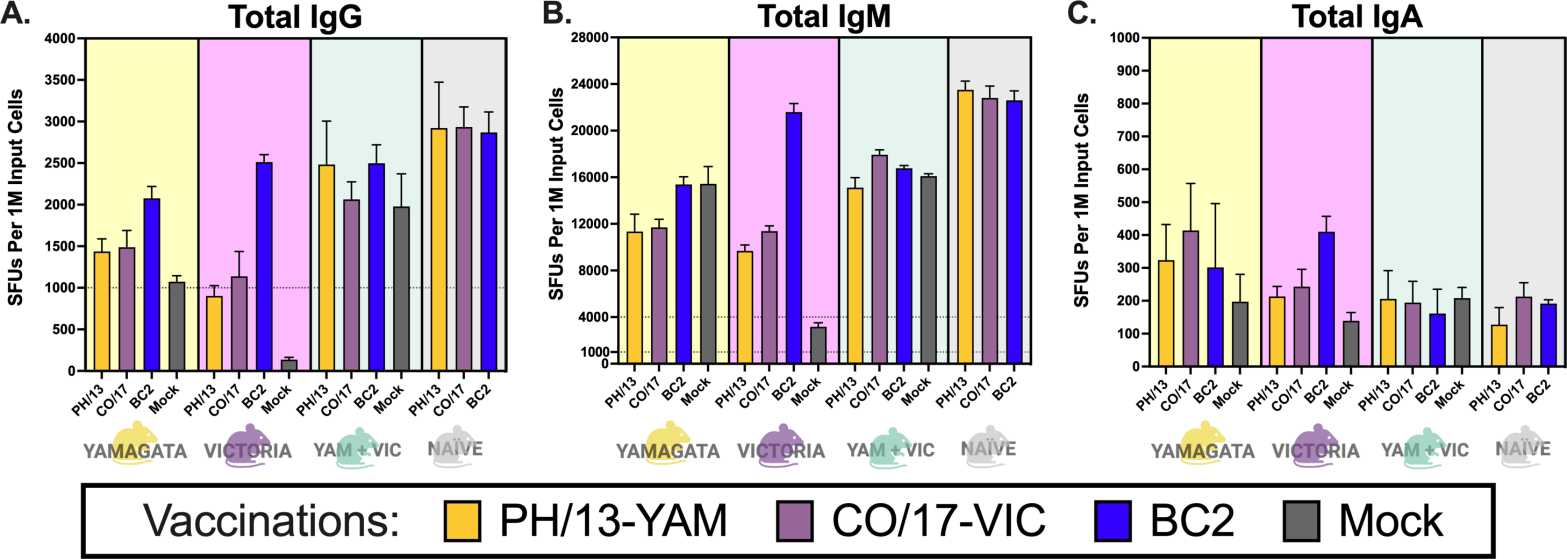
Total Ig secretion from mouse splenocytes with different immunological backgrounds. Three-Color FluoroSpots for IgG (**A**), IgM (**B**), and IgA (**C**) were run using splenocytes from mice pre-immunized with B/SH/02-YAM (yellow background) or B/HK/01-VIC (violet background), or B/SH/02-YAM + B/HK/01-VIC (light blue background), or mice that were influenza naïve (gray background). Mice were vaccinated with either B/PH/13 (orange bars), B/CO/17 (purple bars), BC2 (blue bars), or adjuvanted PBS mock (gray bars). Plates were coated with anti-mouse Igκ + anti-mouse Igλ for total Ig. Samples were run in triplicate, and each bar represents the mean SFUs of the three replicates with an error bar for standard deviation. Dotted lines are added as visual aids to show the *y*-axis limit for IgA (1,000 SFUs) and IgG (4,000 SFUs). Statistical analyses were performed on the SFUs using ordinary one-way ANOVA with Tukey’s multiple comparison test (File S4).

To better assess the immunoglobulin isotype profiles, FluoroSpot assays were used to identify IgG-, IgM-, or IgA-secreting splenic B cells collected 5–6 days post-boost in a subset of mice (Fig. 3 and 4). These mice were immunized again without adjuvant to preferentially stimulate memory B cell responses over those of naïve B cells. To further enhance memory B cell activation, splenocytes were stimulated *in vitro* with a polyclonal activator. Subsequently, the B cells were exposed to various coating antigens to induce differentiation into ASCs that were either HA-specific (Fig. 3) or non-specific (Fig. 4), each of which were labeled with fluorochrome-conjugated antibodies specific to IgG, IgM, or IgA.

To assess the HA-specific ASCs, splenocytes collected from mice were stimulated with a single inactivated virus representing the historical strains used for pre-immunity, B/SH/02 (B/YAM) or B/HK/01 (B/VIC), or the modern strains used for vaccination, B/PH/13 (B/YAM) or B/CO/17 (B/VIC) (Fig. 3). Pre-immune mice had, in general, similar patterns in the number of anti-HA ASCs against the two B/YAM viruses with a higher percentage of cells secreting either IgG or IgA compared to IgM (Fig. 3A and C). In contrast, pre-immune mice had, in general, a higher number of IgM-secreting cells in response to the two B/VIC viruses (Fig. 3B and D). In addition, naïve mice vaccinated with any of the three HA proteins had a higher number of IgM-secreting cells regardless of the virus used for stimulation (Fig. 3). Regardless of pre-immunity, splenocytes from mice vaccinated with B/PH/13-YAM HA had a high percentage of IgG-secreting cells against the two lineage-matched B/YAM viruses (Fig. 3A and C), with significantly more (*P* < 0.0001) IgG-secreting cells when stimulated with B/PH/13 (B/YAM) virus than mice vaccinated with the other immunogens (Fig. 3C). In contrast, splenocytes from mice vaccinated with B/CO/17-VIC HA had a high percentage of IgG-secreting cells only against the B/CO/17 (B/VIC) virus (Fig. 3D and H). Interestingly, this group of B/CO/17-VIC HA-vaccinated mice had the highest percentage of IgG-secreting cells (66%) in B/YAM pre-immune mice (Fig. 3H), with significantly higher numbers of IgG-secreting cells than B/PH/13-YAM HA (*P* < 0.01) and BC2 HA (*P* < 0.05) (Fig. 3D). However, these B/YAM pre-immune mice vaccinated with B/CO/17-VIC HA also had the lowest percentage of IgM-secreting cells (9%) compared to other vaccinated mice (Fig. 3D and H). Similarly, the B/PH/13-YAM HA-vaccinated mice that were pre-immunized with virus from the opposite lineage had high levels of IgG-secreting cells (56%), but with a higher number of IgM-secreting cells (25%) (Fig. 3C and G). Splenocytes from mice pre-immune to B/VIC and then vaccinated with BC2 HA had, non-statistically, the highest number of IgG-secreting cells against the B/VIC viruses compared to other pre-immunized mice (Fig. 3B and D). BC2 HA-vaccinated mice had significantly higher numbers of IgM-secreting cells than mice immunized with the wild-type HA vaccines in many cases, but this was most prominent (*P* < 0.0001) in the splenocytes from mice pre-immune to the B/VIC lineage that were then stimulated with B/HK/01 (B/VIC) virus (Fig. 3B). Lastly, mock-vaccinated mice pre-immune to B/VIC had IgA-secreting cells comprising 66%–79% of their total SFUs (Fig. 3E through H). These B/VIC pre-immune mock-vaccinated mice had a significantly higher (*P* < 0.001) number of IgA-secreting cells compared to each of the HA-vaccinated mice when stimulated against either of the B/YAM viruses (Fig. 3A and C), and a significantly higher (*P* < 0.001) number of IgA-secreting cells in mice vaccinated with wild-type HA vaccines when stimulated with B/HK/01 (B/VIC) virus (Fig. 3B). Splenocytes from B/VIC pre-immune mock-vaccinated mice did not have a significantly higher number of IgA-secreting cells than BC2 HA-vaccinated mice when stimulated with B/HK/01 (B/VIC) virus, but they did have a significantly higher (*P* < 0.05) number of IgA-secreting cells than BC2 HA-vaccinated mice when stimulated with B/CO/17 (B/VIC) virus (Fig. 3D).

To verify that low antigen-specific responses were not due to non-functional splenic B cells, total immunoglobulin secretion was also assessed (Fig. 4). Splenocytes were plated in wells coated with anti-mouse Igκ and anti-mouse Igλ to stimulate ASCs from the entire B cell population that were then labeled for IgG, IgM, or IgA. In general, mice vaccinated with any of the vaccines had statistically similar IgG ASCs regardless of pre-immune background (Fig. 4A). However, mice with single pre-immune backgrounds and vaccinated with wild-type HA vaccines had a significantly fewer number of ASCs compared to dual pre-immune mice or naïve mice vaccinated with the same HA vaccine (Fig. 4A). B/VIC pre-immunized mice vaccinated with BC2 HA had significantly higher (*P* < 0.0001) ASCs than mice vaccinated with B/PH/13-YAM HA or B/CO/17-VIC HA (Fig. 4A). Similar results were observed for anti-IgM, as mice pre-immunized with B/VIC and vaccinated with BC2 HA had significantly higher (*P* < 0.0001) ASCs than mice vaccinated with B/PH/13-YAM HA or B/CO/17-VIC HA (Fig. 4B). Additionally, B/YAM pre-immune mice that were mock-vaccinated or vaccinated with BC2 HA had significantly higher IgM-specific ASCs than mice vaccinated with B/CO/17-VIC HA or B/PH/13-YAM HA (Fig. 4B). Naïve vaccinated mice had statistically higher (*P* < 0.0001) IgM ASCs following vaccination as mice pre-immunized with one or two influenza B viruses (Fig. 4B). Mice pre-immune to both influenza B viruses and vaccinated with a wild-type HA vaccine had a significantly higher (*P* < 0.001) number of IgM ASCs compared to vaccinated mice pre-immune to a single influenza B virus (Fig. 4B). In contrast, mice vaccinated with BC2 HA had a significantly higher (*P* < 0.0001) number of IgM ASCs when pre-immune to the B/VIC virus versus when pre-immune to B/YAM or the combination of both viruses (Fig. 4B). Lastly, B/VIC pre-immune mice that were vaccinated with BC2 HA had a statistically higher (*P* < 0.05) number of IgA-specific ASCs than B/VIC pre-immune mock-vaccinated mice, but no other statistical significance was observed for anti-IgA (Fig. 4C).

The extent and type of immune response varied overall based on prior immunity and vaccine antigen. Mice pre-immunized with a single virus and vaccinated with wild-type vaccines had less total immunoglobulin production compared to dual pre-immune mice or naïve mice vaccinated with the same HA vaccine (Fig. 4A). However, these same mice generally had higher levels of IgG or IgA (Fig. 3), whereas naïve mice and mice pre-immune to both influenza B viruses generally had splenocytes that consisted of a higher number of IgM-secreting cells (Fig. 3). In general, activation induced by either of the two B/YAM viruses resulted in a higher percentage of cells secreting either IgG or IgA (Fig. 3E and G), whereas the response to the B/VIC viruses—especially B/HK/01—elicited a higher number of IgM-secreting cells (Fig. 3F and H). BC2 HA induced robust IgG responses in specific contexts—most notably in B/VIC pre-immune mouse splenocytes stimulated with B/CO/17 (Fig. 3D and H); however, it generally favored the induction of IgM responses. In contrast, wild-type HA vaccines were more effective at enhancing IgG responses, particularly in mice with lineage-matched pre-existing immunity.

### Pre-existing immunity ameliorates disease from IBV infection independent of vaccination

**Fig 5:**
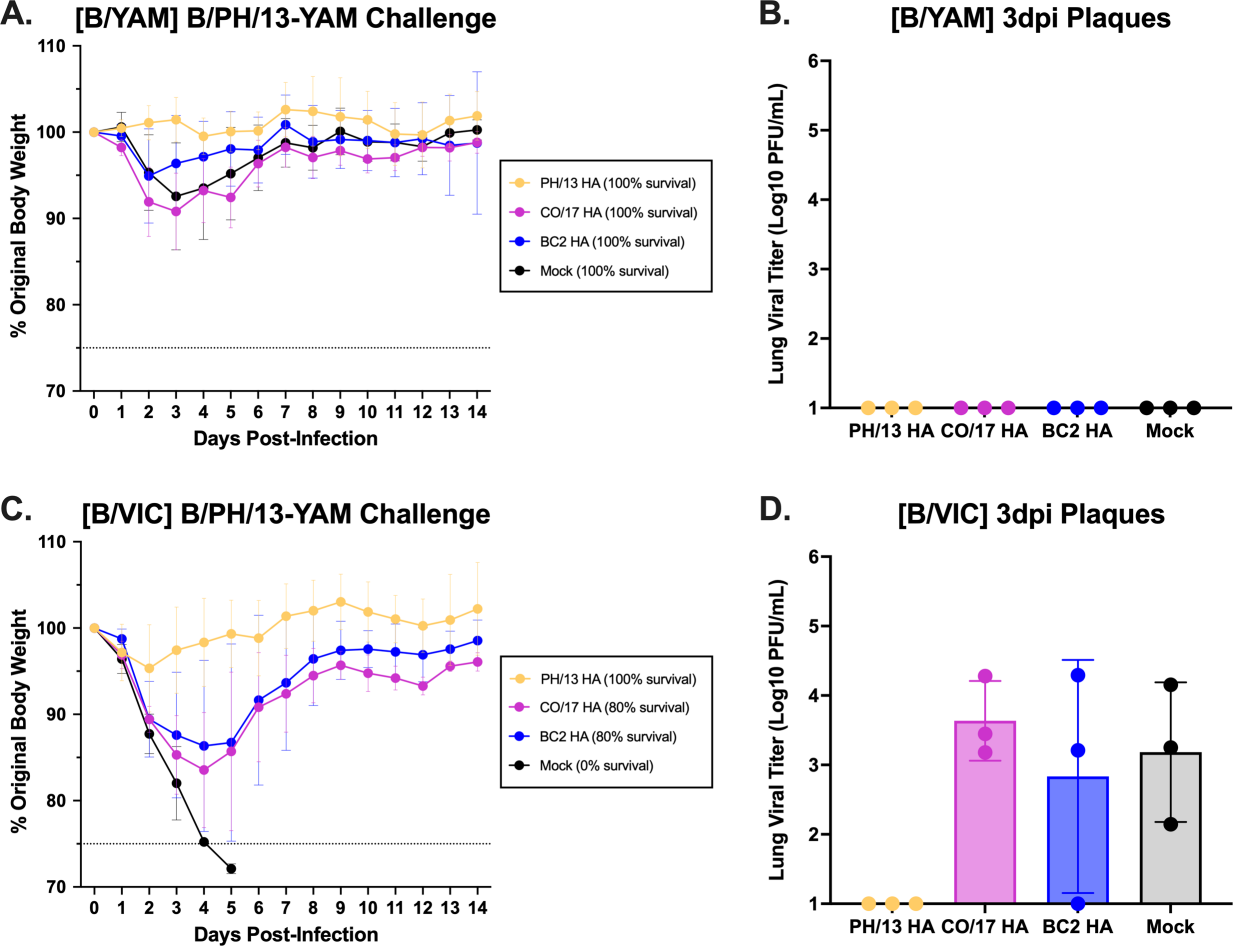
Challenge with B/Phuket/3073/2013 (B/YAM) influenza B virus. Mice pre-immunized to B/SH/02-YAM (**A, B**) or B/HK/01-VIC (**C, D**) were challenged with B/PH/13 (B/YAM) at 3 × 10^5^ PFU/50 µL (**A, C**). Percent of original weight is listed on the *y*-axis and the days post-challenge on the *x*-axis. The dotted line represents 25% wt loss. Percent survival following challenge is listed in the legend. Viral titers were determined from collected lungs 3 dpi via plaque assay (**B, D**). Statistical analyses were performed using ordinary one-way ANOVA with Tukey’s multiple comparison test, but no statistically significant results (*P* ≤ 0.5) were ascertained.

Mice pre-immune to B/YAM or B/VIC viruses were challenged with a B/YAM virus, B/PH/13 (Fig. 5), or a B/VIC virus, B/CO/17 (Fig. S2), and assessed for morbidity or mortality. Mice pre-immunized with B/YAM virus and then vaccinated with B/PH/13-YAM HA had no weight loss (Fig. 5A). Mice pre-immunized with B/YAM virus and then mock-vaccinated or vaccinated with the BC2 or B/CO/17-VIC HA antigens lost on average 5%–8% body weight 2–3 days post-challenge with B/PH/13 (B/YAM) influenza B virus. No B/YAM pre-immune mouse had detectable viral titers 3 days following challenge with B/PH/13 influenza B virus (Fig. 5B). Mice pre-immune to B/VIC virus that were mock-vaccinated and then challenged with B/PH/13 (B/YAM) influenza B virus rapidly lost weight, and all mice died 5 days post-challenge (Fig. 5C). These mice had detectable viral lung titers (10^3.2^ PFU/mL) (Fig. 5D). B/VIC pre-immune mice vaccinated with BC2 HA or B/CO/17-VIC HA and challenged with B/PH/13 (B/YAM) influenza B virus lost on average 15% of their body weight by day 5 post-challenge, but slowly recovered (Fig. 5C) with similar levels of viral lung titers as mock-vaccinated mice (Fig. 5D). In contrast, B/VIC pre-immune mice vaccinated with B/PH/13-YAM HA lost little weight and had no detectable viral lung titers. All mice challenged with B/CO/17 (B/VIC) survived the challenge with little-to-no weight loss and little-to-no detectable virus in their lungs (Fig. S2).

**Fig 6:**
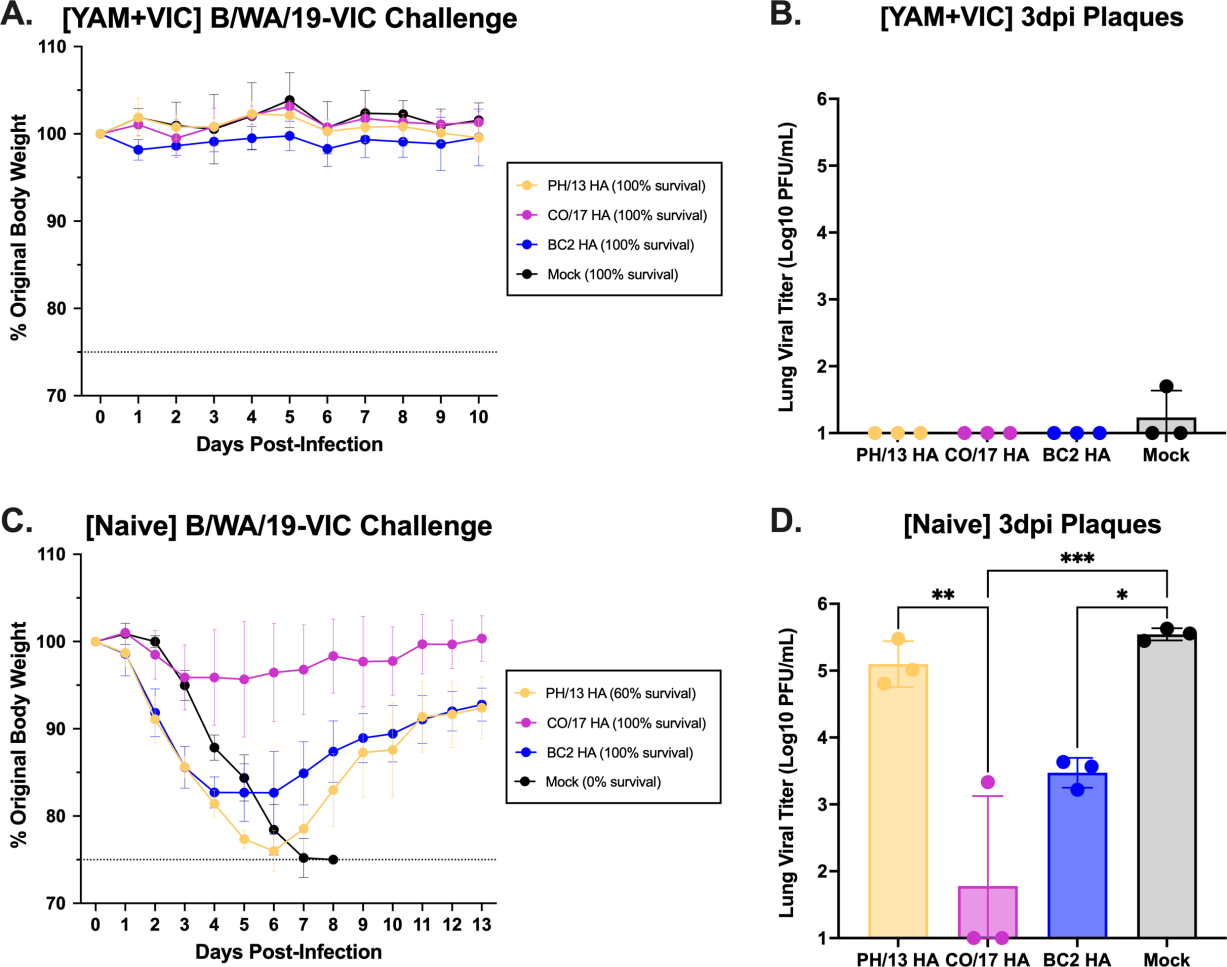
Challenge with B/Washington/2/2019 (B/VIC) influenza B virus. Mice pre-immunized to B/SH/02-YAM and B/HK/01-VIC (**A, B**) or immunologically naïve mice (**C, D**) were challenged with B/WA/19 (B/VIC) virus at 1 × 10^6^ PFU/50 µL (**A, C**). Percent of original weight is listed on the *y*-axis and the days post-challenge on the *x*-axis. The dotted line represents 25% wt loss. Percent survival following challenge is listed in the legend. Viral titers were determined from collected lungs 3 dpi via plaque assay (**B, D**). Statistical analyses were performed using ordinary one-way ANOVA with Tukey’s multiple comparison test. *P ≤ 0.05, **P ≤ 0.01, ***P ≤ 0.001, and ****P ≤ 0.0001.

Mice pre-immune to both B/SH/02 (B/YAM) and B/HK/01 (B/VIC) influenza B viruses were challenged with the drifted B/WA/19 (B/VIC) influenza B virus (Fig. 6A). All of these pre-immune mice vaccinated with any of the three HA antigens lost no weight and had no detectable lung virus (Fig. 6A and B). In addition, there was no weight loss in mock-vaccinated pre-immune mice challenged with B/WA/19 (B/VIC) virus.

In contrast, naïve mice that were mock-vaccinated and challenged with B/WA/19 (B/VIC) influenza B virus lost 25% of their body weight and succumbed to infection between days 7 and 8 post-challenge (Fig. 6C), with high levels of virus detected in their lungs (10^5.5^ PFU/mL) (Fig. 6D). Naïve mice vaccinated with BC2 HA or B/PH/13-YAM HA lost, on average, 15%–25% original body weight between days 5 and 7 post-challenge (Fig. 6C). Mice vaccinated with B/PH/13-YAM HA had viral lung titers statistically similar to mock-vaccinated mice, whereas mice vaccinated with BC2 HA had viral titers that were two logs lower (10^3.5^ PFU/mL) (Fig. 6D). Mice vaccinated with B/CO/17-VIC HA lost less than 5% weight and had low viral lung titers (10^1.7^ PFU/mL), with undetectable viral titers in two of these mice (Fig. 6D).

Pre-existing immunity strongly influenced protection from influenza B virus infection. Pre-immunity to a single lineage conferred full protection against homologous challenge, but not always against heterologous challenge. B/VIC pre-immune mice challenged with the B/PH/13 (B/YAM) virus had complete protection if vaccinated with B/PH/13-YAM HA, but only partial protection when vaccinated with B/CO/17-VIC HA or BC2 HA (Fig. 5C). Unfortunately, the B/CO/17 (B/VIC) virus was not very pathogenic to any pre-immunized mice to better compare the vaccines (Fig. S2). Naïve mice challenged with the drifted B/WA/19 (B/VIC) virus had complete protection if vaccinated with B/CO/17-VIC HA or BC2 HA, but only partial protection if vaccinated with B/PH/13-YAM HA (Fig. 6C). Dual pre-immunity conferred full protection from B/WA/19 (B/VIC) infection regardless of vaccine, highlighting the benefit of prior exposure to both influenza B lineages (Fig. 6A).

## DISCUSSION

Previous exposures to influenza viruses shape the immune responses upon subsequent viral re-exposures (47). This concept, known as “original antigenic sin,” is described as an imprint by the original virus infection governing future antibody responses to drifted influenza virus infections (47). Potential negative effects of these antigenic interactions, such as “antigenic seniority” (48), may recall responses targeted toward epitopes that have since undergone antigenic drift (48, 49). While protective antibody titers to the primary infection strain are maintained, there is low antibody binding to new strains of influenza (50–52). Over time, this back-boosting of previous memory B cells will narrow antibody repertoires toward immunodominant, but non-protective epitopes, instead of broadening the repertoire to recognize a wide variety of epitopes on diverse viral strains (17, 52). In contrast, immune imprinting may positively contribute to protection against pre-pandemic influenza subtypes, such as H5N1 and H7N9 (53), as well as influenza strains during previous H1N1 pandemics in 1977 and 2009 (54). Whether pre-existing immunity can facilitate a heightened antibody response or can negatively interfere with vaccine-mediated protection is not entirely clear. In this study, a broadly reactive influenza hemagglutinin vaccine antigen was tested in mice with and without pre-existing immunity. Naïve mice vaccinated with the BC2 HA had little-to-no HAI titers against the panel of influenza B viruses. However, mice with pre-existing influenza B immunity vaccinated with BC2 HA proteins activated memory B cells and elicited protective antibodies with HAI activity against a panel of influenza B viruses representing both IBV lineages.

Lineage-specific immunological imprinting with influenza B viruses has not been well characterized. In this study, mice with pre-immunity to a historical B/YAM influenza B virus preferentially boosted antibodies with HAI activity against the panel of B/YAM viruses, and mice with pre-immunity to a historical B/VIC influenza B virus preferentially boosted antibodies with HAI activity against a panel of B/VIC viruses. B/VIC pre-immune mice vaccinated with B/PH/13-YAM HA protein had antibodies with HAI activity against viruses from both IBV lineages. Whereas B/YAM pre-immune mice vaccinated with B/CO/17-VIC HA had low HAI titers against both lineages. Phylogenetically, the BC2 HA sequence is located between viruses in the B/YAM and B/VIC lineages and shares amino acids in antigenic regions associated with each lineage (Fig. S3). However, the BC2 HA has sequence homology closer to B/VIC viruses, with more B/VIC-like sites in regions where the two lineages differ the most, particularly the 150-loop, the 160-loop, and 190-helix (Fig. S3). As such, when pre-immunized with a B/VIC virus, BC2 HA-vaccinated mice generally elicited HAI activity against B/VIC viruses as mice vaccinated with B/CO/17-VIC HA. However, B/YAM pre-immune mice vaccinated with BC2 HA also elicited antibodies with HAI activity against the B/YAM panel of influenza B viruses. BC2 still retains B/YAM-specific epitopes located in regions such as the 120-loop and surrounding areas (Fig. S3). Moreover, the BC2 HA sequence was designed using HA amino acid sequences from influenza B viruses isolated from people between 1940 and 2011 (41), so it was not unexpected to see lower HAI and B cell responses to viruses isolated between 2013 and 2019. However, BC2 HA-vaccinated mice had similar HAI titers and B cell responses against these future drifted influenza B viruses as mice vaccinated with the B/PH/13-YAM HA and B/CO/17-VIC HA proteins.

The prevailing hypothesis behind this study was that a broadly reactive HA would be capable of activating memory B cells from more diverse repertoires compared to wild-type HA vaccines. On average across the different pre-immune backgrounds, BC2 HA stimulated the production of higher levels of Ig. However, there were higher levels of IgM-secreting B cells in splenocytes regardless of the vaccination antigen. Canonically, antigen-activated memory B cells are primarily associated with IgG following clonal expansion, somatic hypermutation, and class-switching to replace surface IgM expression with IgG (or another isotype) (2, 7–9). IgM is more associated with short-term, innate-like qualities as the initial antibody of the adaptive immune system (5, 6). However, antigen-specific IgM-secreting B cells from the mouse spleen are involved in long-term immunity and have the ability to protect against influenza virus independent of IgG, as demonstrated in a lethal H1N1 viral challenge following depletion of IgG-positive mouse B cells following treatment with αCD40L antibody (7). Isotype analysis in serum showed much higher levels of IgG compared to IgM and IgA (Fig. S1). However, these serum antibodies were detected 2–3 weeks post-vaccination, whereas the splenocytes used in the FluoroSpot assay were collected 5–6 days post-vaccination. The half-life of circulating antibodies following secretion from B cells is only 5 days for IgM, 6 days for IgA, and 23 days for IgG (2). Additionally, IgG makes up ~75% of the antibody found in serum, whereas IgA consists of ~15% and IgM consists of ~10% (4). Therefore, samples collected 2–3 weeks post-vaccination would be expected to be predominately IgG, as we show in this study via ELISA, but samples collected 5-6 days following a vaccination boost might be higher in IgA and IgM, as we show in the splenocytes for some groups of vaccinated mice (Fig. 3).

The BC2 HA sequence has more diverse IBV HA epitopes, as well as conserved epitopes, across the influenza B virus history. Secretory IgM can bind multiple epitopes on the same antigen or across different antigens due to its pentameric structure with 10 antigen-binding sites (55–57). While this polyreactive nature is associated more with multivalent polysaccharide vaccines (58, 59) from IgM secreted by B-1 cells produced during fetal development (55, 56), this polyreactivity has additionally been shown for anti-influenza HA antibodies that target more conserved epitopes (60, 61) and from IgM secreted by B-2 cells developed later in life (62, 63). Therefore, the increased production of IgM-secreting cells elicited by the broadly reactive BC2 HA supports this previous research and adds to it through the inclusion of influenza B virus.

IgM works in concert with IgG to neutralize pathogens, as demonstrated in IgM-deficient mice (64–66). Likewise, in this study, the elicitation of predominantly IgG-secreting cells does not automatically dictate antibody neutralization of influenza B viruses. The highest percentage of IgG-secreting cells was observed following stimulation with a virus matching the lineage-specific HA used for vaccination in an immunologically different IBV lineage background. But while the B/PH/13-YAM HA-vaccinated mice pre-immunized to B/VIC virus (56% IgG, 25% IgM, 20% IgA) elicited high HAI titers against B/PH/13 (B/YAM) virus following vaccination, the B/CO/17-VIC HA-vaccinated mice pre-immunized to a B/YAM virus (66% IgG, 9% IgM, 24% IgA) did not elicit high HAI titers post-vaccination. A higher percentage of IgG-secreting cells is a good indicator for antigen-specific neutralization, but ultimately, a diverse set of elicited HA-specific B cells expressing IgG, IgM, and IgA all contribute to the overall neutralization of viruses.

Humans have a lifetime of different infections and vaccinations that contribute to a robust and diverse repertoire of memory B cells (9, 52, 53, 67–71). And while animal models cannot perfectly recapitulate the complexities of the human condition, using pre-immune animals can help provide additional insight into how memory B cells may be activated upon future exposures. These animal models allow for the ability to test different immune histories, assess novel vaccinations, and explore different B cell populations in peripheral tissues. However, animal models also have limitations of their own. Spleens are a major reservoir for memory B cells in mice and humans; however, the MZ B cells, which express high levels of IgM, differ between mice and humans (13, 72–74). Human MZ B cells recirculate freely to other sites outside the spleen, and these somatically mutated, circulating IgM-positive memory B cells appear to be identical to the MZ B cells that reside in the spleen (74). While murine MZ B cells are important for the production of antibodies against blood-borne antigens, these murine MZ B cells use an adhesion G-protein-coupled receptor, CD97, for retention in the spleen (75). Murine splenocytes can function in antigen delivery to follicular B cells (72, 76); however, characterizing B cell activation by influenza vaccines would be more relevant with a population of circulating B cells. Therefore, future studies utilizing other mammalian splenocytes or peripheral blood mononuclear cells (PBMCs) to measure memory B cell responses may be warranted to better assess the impact that different pre-existing immunities have on establishing diverse memory B cell repertoires for vaccine-induced recall. Utilizing other mammalian models may also address potential issues with the B/VIC pre-immune mouse model, since almost all (99.7%) of these mice had HAI titers against B/HK/01, but only 62% of mice had seroprotective HAI levels. When establishing this model, doses of B/HK/01 that were too high inadvertently killed some mice, whereas doses of virus that were lower failed to elicit any seroconversion. In contrast, all mice pre-immunized to B/SH/02 (B/YAM) influenza virus had seroprotective HAI titers against B/SH/02. Additionally, the B/VIC (B/HK/01) virus did not elicit any pre-vaccination titers toward the B/YAM virus panel, but our previously published pre-immune ferret model using the same B/VIC influenza B virus elicited seroprotective HAI titers against the B/YAM influenza B viruses, as well as high HAI titer against B/VIC influenza B viruses (41). For mice with pre-existing immunity to both B/SH/02 (B/YAM) and B/HK/01 (B/VIC), infecting mice with both strains reduced morbidity and eliminated the mortality caused by B/HK/01 (B/VIC) influenza virus alone. However, immunodominance was observed against the B/YAM lineage influenza B virus with higher HAI titers than the B/VIC lineage virus. To alter this immunodominance, these two viruses were used for infection in different ratios with the goal of equalizing the antibody responses against the viruses from both lineages (Fig. S4). Nevertheless, average HAI titers against B/SH/02 (B/YAM) were still ~2-fold higher than B/HK/01 (B/VIC) with the doses used in this study, despite giving B/HK/01 (10^4^ PFU/50 µL) at a higher dose than B/SH/02 (10^2.5^ PFU/50 µL).

Overall, this study demonstrated a lineage specificity for immunological imprinting with influenza B viruses. Imprinting to one lineage can, in some cases, negatively influence vaccine-elicited responses by wild-type HA antigens. However, the use of broadly reactive HA antigens as vaccines can elicit effective antibody and B cell immunity in animals with diverse pre-existing immunity. This may translate into a pool of broadly reactive IgM-positive memory B cells that are similar to naïve B cells, but with the ability to rapidly respond to antigen stimulation (13). The BC2 HA may utilize this process to activate long-term IgM-secreting B cells to neutralize a broad panel of viruses.

## Data Availability

All data are provided in the supplemental material. For HAI data used to generate Fig. 2, Tables 1 and 2, and Fig. S4, see File S1. For ELISA data used to generate Fig. S1, see File S2. For FluoroSpot data used to generate Fig. 3 and 4, see File S3. For statistical analysis related to Fig. 3 and 4, see File S4. For viral challenge weight loss, mortality, and plaque data related to Fig. 5 and 6 and Fig. S2, see File S5.

## SUPPLEMENTAL MATERIAL

File S1 jvi.00705-25-s0001.xlsx HAI data used to generate Fig. 2, Table 1, Table 2, and Fig. S4.

File S2 jvi.00705-25-s0002.xlsx ELISA data used to generate Fig. S1.

File S3 jvi.00705-25-s0003.xlsx FluoroSpot data used to generate Fig. 3 and 4.

File S4 jvi.00705-25-s0004.xlsx Statistical analysis related to Fig. 3 and 4.

Fig. S1 jvi.00705-25-s0005.tiff Isotype ELISAs on post-vaccination serum.

Fig. S2 jvi.00705-25-s0006.tiff Challenge with B/Colorado/6/2017 (B/VIC) influenza B virus.

Fig. S3 jvi.00705-25-s0007.tiff BC2 HA sequence alignment with wild-type IBVs.

Fig. S4 jvi.00705-25-s0008.tiff Mixed pre-immunity in mice.

Supplemental legends jvi.00705-25-s0009.docx Legends for Fig. S1 to S4.

File S5 jvi.00705-25-s0010.xlsx Viral challenge weight loss and plaque data.

ASM does not own the copyrights to Supplemental Material that may be linked to, or accessed through, an article. The authors have granted ASM a non-exclusive, world-wide license to publish the Supplemental Material files. Please contact the corresponding author directly for reuse.
