# Supplementary figures and images for: Breadth of antibody activity elicited by an influenza B hemagglutinin vaccine is influenced by pre-existing immune responses to influenza B viruses
Source: J Virol. 2025 Jul 15;99(8):e00705-25. doi: 10.1128/jvi.00705-25 (PMC12363200; doi:10.1128/jvi.00705-25)

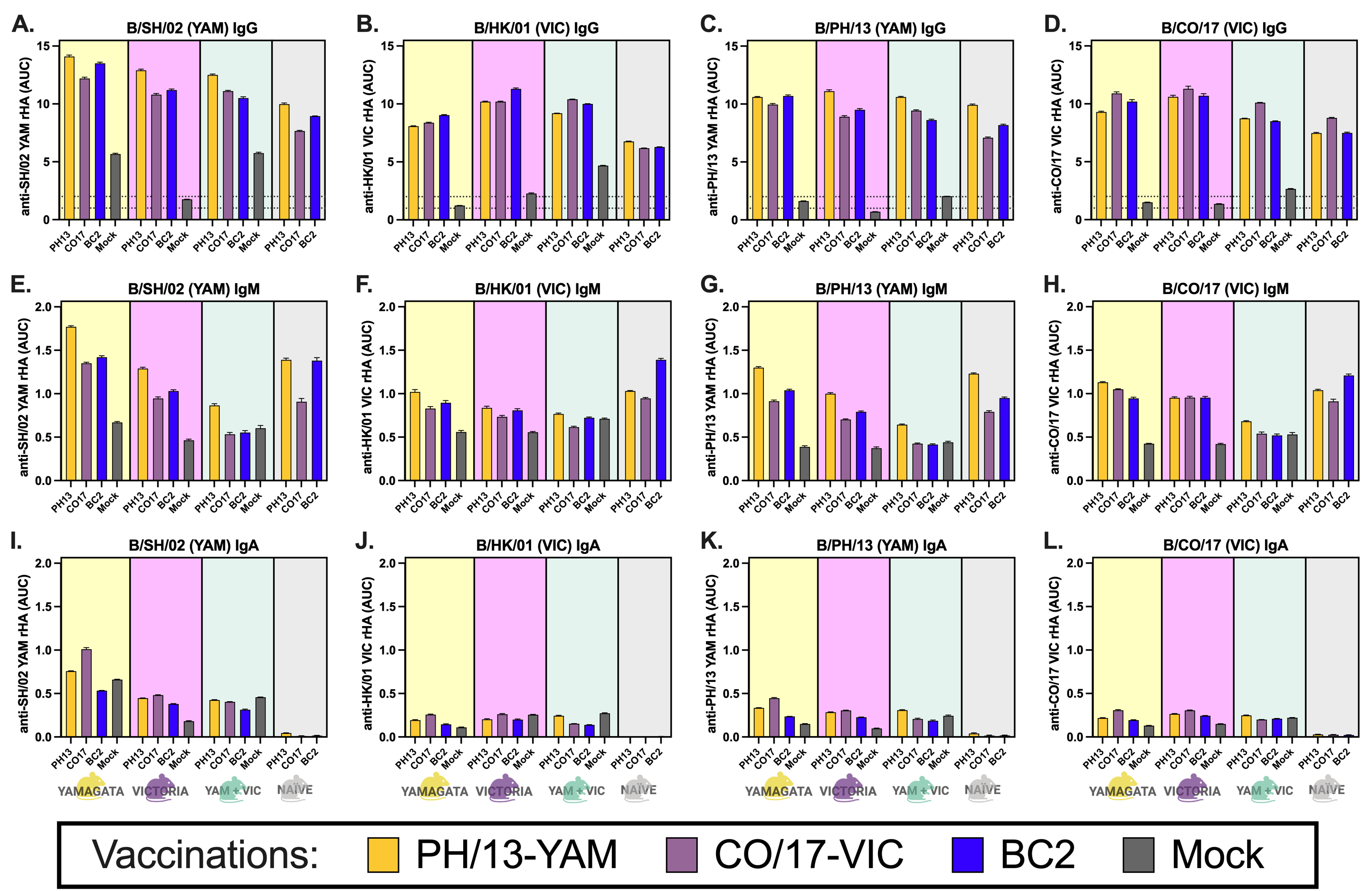

Supplement: Fig. S1 — Isotype ELISAs on post-vaccination serum. [file jvi.00705-25-s0005.tiff]

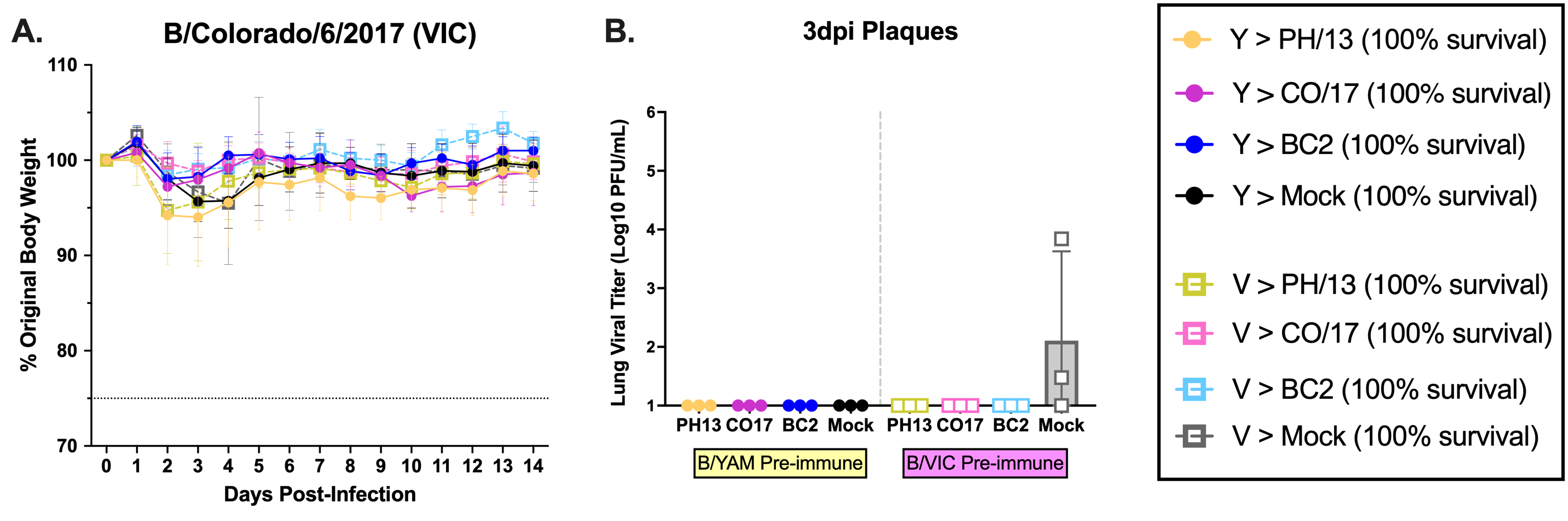

Supplement: Fig. S2 — Challenge with B/Colorado/6/2017 (B/VIC) influenza B virus. [file jvi.00705-25-s0006.tiff]

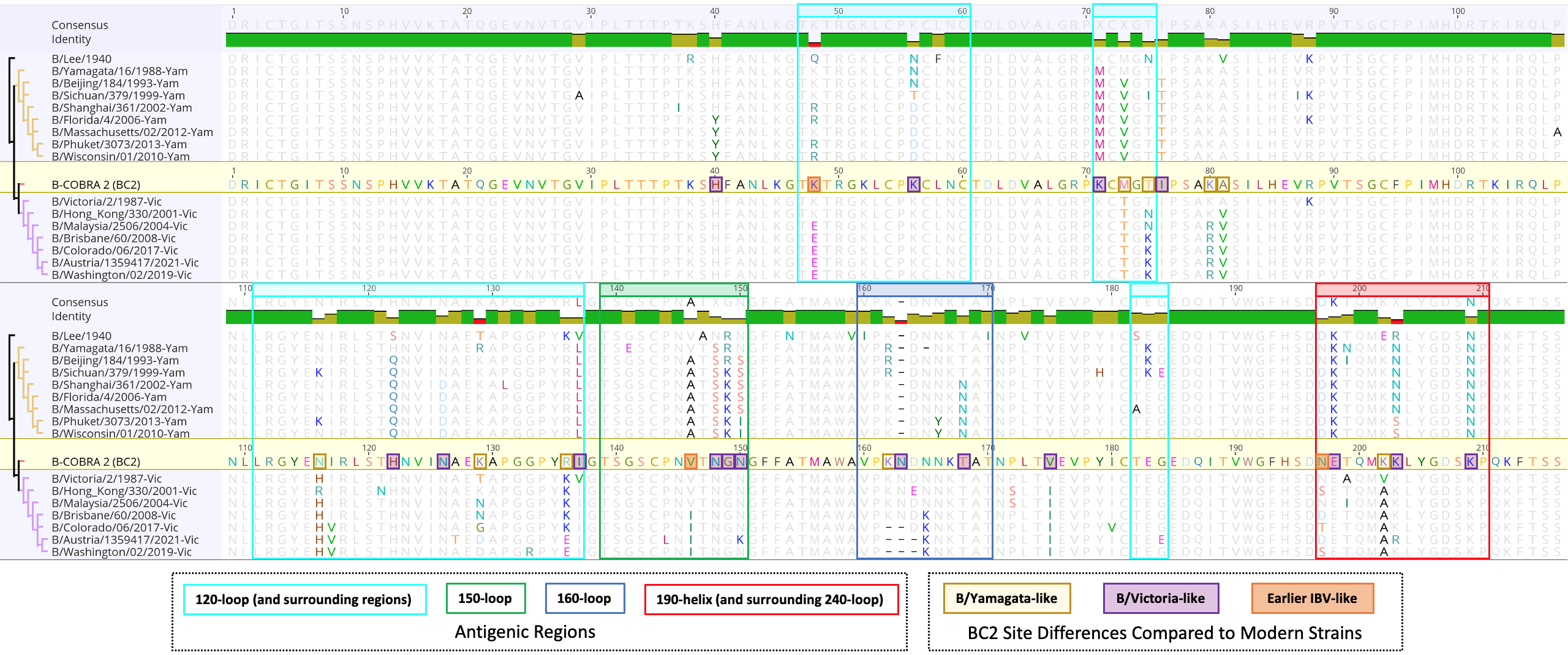

Supplement: Fig. S3 — BC2 HA sequence alignment with wild-type IBVs. [file jvi.00705-25-s0007.tiff]

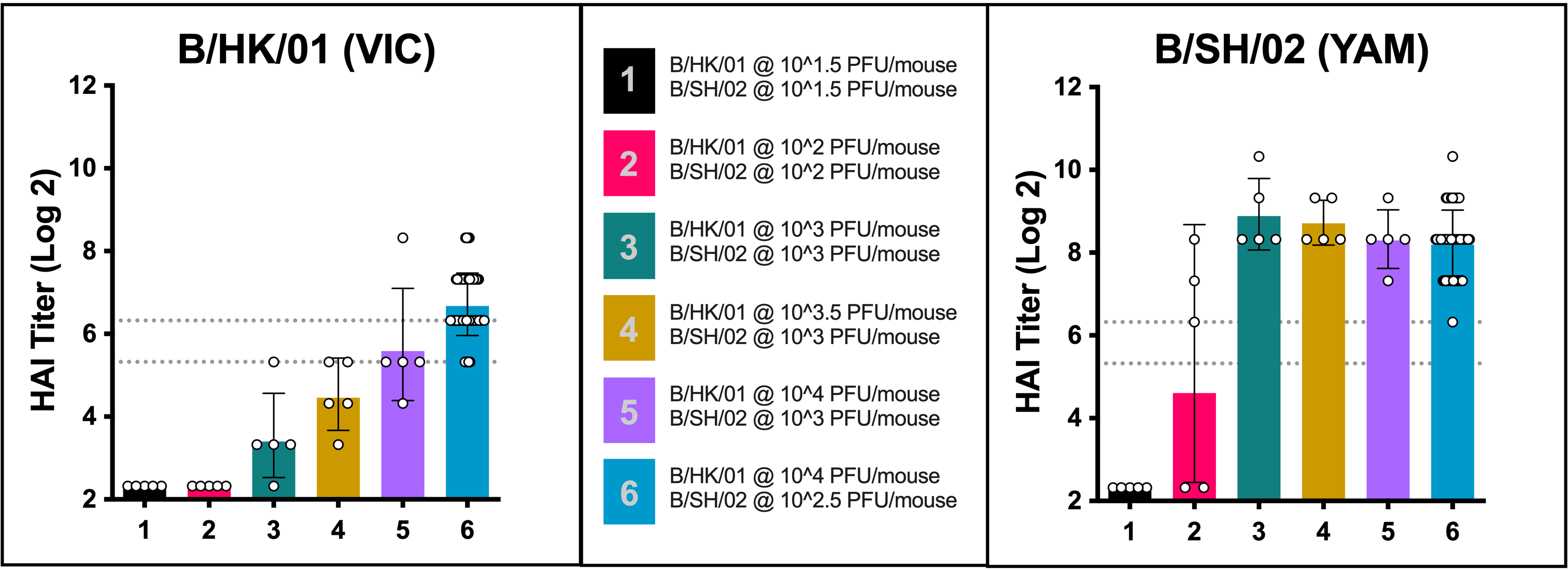

Supplement: Fig. S4 — Mixed pre-immunity in mice. [file jvi.00705-25-s0008.tiff]
