# Supplementary material for: Breadth of antibody activity elicited by an influenza B hemagglutinin vaccine is influenced by pre-existing immune responses to influenza B viruses
Source: J Virol. 2025 Jul 15;99(8):e00705-25. doi: 10.1128/jvi.00705-25 (PMC12363200; doi:10.1128/jvi.00705-25)
Supplement: Supplemental legends — Legends for Fig. S1 to S4. [file jvi.00705-25-s0009.docx]

**Figure S1. Isotype ELISAs on Post-Vaccination Serum.**ELISAs measuring anti-IgG (**A-D**), anti-IgM (**E-H**), and anti-IgA (**I-L**) were run using post-vaccination serum from mice pre-immunized with B/SH/02-YAM (yellow background), or B/HK/01-VIC (violet background), or B/SH/02-YAM + B/HK/01-VIC (light blue background), or mice that were influenza naïve (gray background).  Mice were vaccinated with either B/PH/13 (orange bars), B/CO/17 (purple bars), BC2 (blue bars), or adjuvanted PBS Mock (gray bars).  Plates were coated with rHA proteins representing the pre-immune strains, B/SH/02-YAM (**A, E, I**) and B/HK/01-VIC (**B, F, J**), or the more modern WT comparators, B/PH/13-YAM (**C, G, K**) and B/CO/17-VIC (**D, H, K**).  Serum samples were pooled for each vaccination group and run in duplicate.  Linear curves for the two replicates of each group were created in GraphPad Prism to plot O.D. values across two-fold serum dilutions (ranging from 500 to 512,000 for IgG and from 50 to 12,800 for IgM and IgA).  Total area-under-the-curve (AUC) and standard error were calculated using GraphPad Prism, and the values were subsequently plotted as bars, with the total AUC represented by the bar, and the standard error of the linear curve shown as error bars above each bar.

**Figure S2. Challenge with B/Colorado/6/2017 (B/VIC) influenza B virus.** Mice pre-immunized to B/SH/02-YAM (**A-B**) or B/HK/01-VIC (**C-D**) were challenged with B/CO/17 (B/VIC) at 3 x 10^5 PFU/50μL (**A, C**). The percent original weight is listed on the y-axis and the days post-challenge on the x-axis. The dotted line represents 25% weight loss. Percent survival following challenge is listed in the legend. Viral titers were determined from collected lungs 3 days post-infection (dpi) via plaque assay (**B, D**). Statistical analyses were performed using ordinary one-way ANOVA with Tukey's multiple comparison test, but no statistically significant results (p < 0.5) were ascertained.

**Figure S3. BC2 HA sequence alignment with wild-type IBVs.** Sequence alignment of various IBV HAs, created using Geneious version 2020.1 with Muscle 3.8.425 followed by Geneious Tree Builder (Bootstrap resampling with 100 replicates and B/Lee/1940 as an outgroup). B/Yamagata-lineage viruses grouped in yellow on the tree. B/Victoria-lineage viruses grouped in violet. Major antigenic regions outlined for the 120-loop and surrounding regions (cyan), 150-loop (green), 160-loop (blue) and the 190-helix and surrounding 240-loop (red). BC2 HA is highlighted in the middle as the reference sequence, and the residue colors indicate differences from the BC2 HA sequence. Certain amino acids are highlighted in the BC2 HA sequences as being characteristic of a B/Yamagata-like residue (yellow box), a B/Victoria-like residue (violet box), or an earlier IBV-like residue no longer associated with more modern strains (orange box). All sequences begin after the 15 amino acid signal sequence.

**Figure S4. Mixed pre-immunity in mice.** BALB/c mice were intranasally infected with a 50μL mixture of B/HK/01 (B/VIC) and B/SH/02 (B/YAM) diluted in sterile PBS at different doses. Serum was collected 4-6 weeks later, and HAI was performed against each representative strain. Groups 1-5 contain an n-value of 5 mice, and group 6, the dose used for the overall study, contains an n-value of 56 mice (n=14/vaccination group). Log2 HAI titers for individual mice are shown as scatter dot plots with bars representing the geometric mean titer of each group and error bars indicating the geometric standard deviation. Dotted lines show an HAI titer of 1:40 and 1:80.
